# Eggshell membrane powder ameliorates intestinal inflammation by facilitating the restitution of epithelial injury and alleviating microbial dysbiosis

**DOI:** 10.1038/srep43993

**Published:** 2017-03-08

**Authors:** Huijuan Jia, Manaka Hanate, Wanping Aw, Hideomi Itoh, Kenji Saito, Shoko Kobayashi, Satoshi Hachimura, Shinji Fukuda, Masaru Tomita, Yukio Hasebe, Hisanori Kato

**Affiliations:** 1 Corporate Sponsored Research Program “Food for Life,” Organization for Interdisciplinary Research Projects, The University of Tokyo, Tokyo, Japan; 2 Department of Applied Biological Chemistry, Graduate School of Agricultural and Life Sciences, The University of Tokyo, Tokyo, Japan; 3 Institute for Advanced Biosciences, Keio University, Yamagata, Japan; 4 Bioproduction Research Institute, National Institute of Advanced Industrial Science and Technology (AIST) Hokkaido, Sapporo, Japan; 5 Research Center for Food Safety, Graduate School of Agricultural and Life Sciences, The University of Tokyo, Tokyo, Japan; 6 Research Center for Food Safety and Department of Applied Biological Chemistry, Graduate School of Agricultural and Life sciences, The University of Tokyo, Tokyo, Japan; 7 ALMADO Inc., Tokyo, Japan

## Abstract

Gut microbiota is an essential factor in the shaping of intestinal immune system development and driving inflammation in inflammatory bowel disease (IBD). We report the effects and microbe-host interactions underlying an intervention using fine powder of eggshell membrane (ESM) against IBD. ESM attenuated lipopolysaccharide-induced inflammatory cytokine production and promoted the Caco-2 cell proliferation by up-regulating growth factors *in vitro*. In a murine model of dextran sodium sulphate-induced colitis, ESM significantly suppressed the disease activity index and colon shortening. These effects were associated with significant ameliorations of gene expressions of inflammatory mediators, intestinal epithelial cell proliferation, restitution-related factors and antimicrobial peptides. Multifaceted integrated omics analyses revealed improved levels of energy metabolism-related genes, proteins and metabolites. Concomitantly, cecal metagenomic information established an essential role of ESM in improving dysbiosis characterized by increasing the diversity of bacteria and decreasing absolute numbers of pathogenic bacteria such as *Enterobacteriaceae* and *E. coli*, as well as in the regulation of the expansion of Th17 cells by suppressing the overgrowth of segmented filamentous bacteria. Such modulations have functional effects on the host; i.e., repairing the epithelium, regulating energy requirements and eventually alleviating mucosal inflammation. These findings are first insights into ESM’s modulation of microbiota and IBD suppression, providing new perspectives on the prevention/treatment of IBD.

Inflammatory bowel disease (IBD) is a complex, multifactorial immunomodulatory disorder that is associated with a genetic predisposition, environmental factors (including dietary habits), the gut environment, intestinal epithelial integrity and the immune system^1^. The precise pathogenesis of IBD has not been fully elucidated, but there is growing evidence suggesting that IBD is a dysregulated mucosal immune response to food antigens. This response is subjected to modification by a variety of environmental factors, such as pathogens, viruses and gut microbiota imbalance (dysbiosis).

Current therapeutics for IBD aim to counteract the immune dysregulation by inhibiting pro-inflammatory cytokines, T-cell activation and leukocyte adhesion; to restore mucosal barrier function and stimulate epithelial restitution and integrity by different growth factors; as well as to restore the normal bacterial flora with treatments using antibiotics, prebiotics, probiotics or fecal microbiota transplantation^2^,^3^,^4^. The prevention and the control of symptoms for the increasing numbers of IBD patients are highly desirable, but the symptomatic treatment currently used for IBD is accompanied by side effects, and no drugs are able to heal the bowel mucosa. It has been generally accepted that non-pharmaceutical nutritional interventions are the primary therapy for IBD^5^.

One of the natural byproducts of egg processing is the eggshell membrane (ESM), which is usually discarded as an industrial waste, resulting in environmental and financial burdens. Since 2012, when the safety and anti-inflammatory effects of ESM were first reported by Ruff *et al*.^6^, ESM has been shown in human trials to suppress arthritic pain and stiffness as a dietary supplement^7^. We previously obtained nutrigenomic information from *in vivo* and *in vitro* investigations of ESM, and we observed biochemical functions of ESM that counter liver injury and fibrosis. These protective effects of ESM appear to be based on the regulation of proteins and genes that are related to the activation of hepatic stellate cells and fibrogenesis, via a potential novel modulation of the PPARγ-endothelin 1 interaction signaling pathway^8^.

ESM, a “resistant protein” with low digestibility (approx. 46%) according to our previous digestibility test using rats (unpublished data), is a source of non-digestible protein which may have a physiological function similar to that of dietary fiber, to stimulate cecal fermentation and alter the intestinal bacterial composition, the important effective factor for inflammatory colitis. In the present study, we aimed to obtain an accurate and comprehensive understanding of the effects of dietary ESM on IBD prevention and the mechanisms underlying these effects. We identified the actions of ESM and uncovered the potential novel underlying mechanisms in a lipopolysaccharide (LPS)-stimulated *in vitro* model and a dextran sodium sulphate (DSS)-induced colitic murine model by applying a pioneering multifaceted integrated omics approach. We found that ESM not only regulated the cell proliferation and restitution but also ameliorated energy metabolism as well as intestinal microbiota dysbiosis. We attribute these effects to altered host defense and decreased susceptibility of the host to intestinal inflammation.

## Results

### ESM promotes proliferation of intestinal cell and inhibits their LPS-induced expression of major inflammatory cytokines

**Figure 1:**
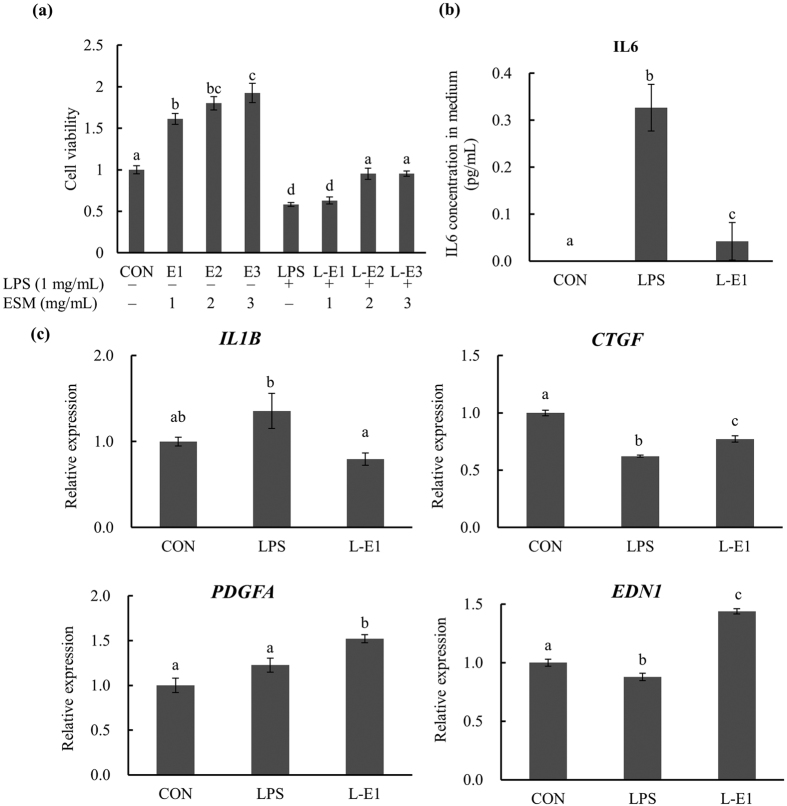
Effects of ESM on LPS-stimulated Caco-2 cells. (**a**) Cell viability, (**b**) IL-6 concentration in medium, (**c**) gene expression in cells. Results are mean ± SE in each group (n = 3). Data with different letters (**a,b,c**) are significantly different at *P* < 0.05 by Dunnett’s test.

To determine the role of ESM in intestinal inflammation, we first addressed its impact on LPS-stimulated Caco-2 cells. After a 24-hr ESM treatment, the cell viability was significantly increased both with and without the LPS stimulation (Fig. 1a). The interleukin (IL)-6 concentration in the medium was significantly lower in LPS + ESM-treated cells compared to LPS-stimulated cells alone (Fig. 1b). The real-time reverse transcription-polymerase chain reaction (RT-PCR) results indicated the following in LPS-treated cells: the expression of genes for *IL-1β* (a vital mediator of the inflammatory response) were significantly down-regulated by the supplementation with ESM; the same supplementation up-regulated the cell proliferation-related factors connective tissue growth factor (*CTGF*) and platelet-derived growth factor alpha (*PDGFA*), and the potent vasoconstrictor peptide endothelin 1 (*EDN1*) (Fig. 1c).

### ESM-supplemented mice displayed ameliorated severity of DSS-induced colitis

Since cell proliferation is associated with intestinal epithelial restitution in injured tissue, we next investigated whether ESM promotes intestinal epithelial restitution and ameliorates the intestinal injury in DSS-induced colitis. Only nonsignificant differences in the total food and water intake before DSS-drinking were observed among the three groups, and in the DSS intake (water intake after DSS administration) between the DSS and D-ESM8 groups (Suppl. Table S3).

**Figure 2:**
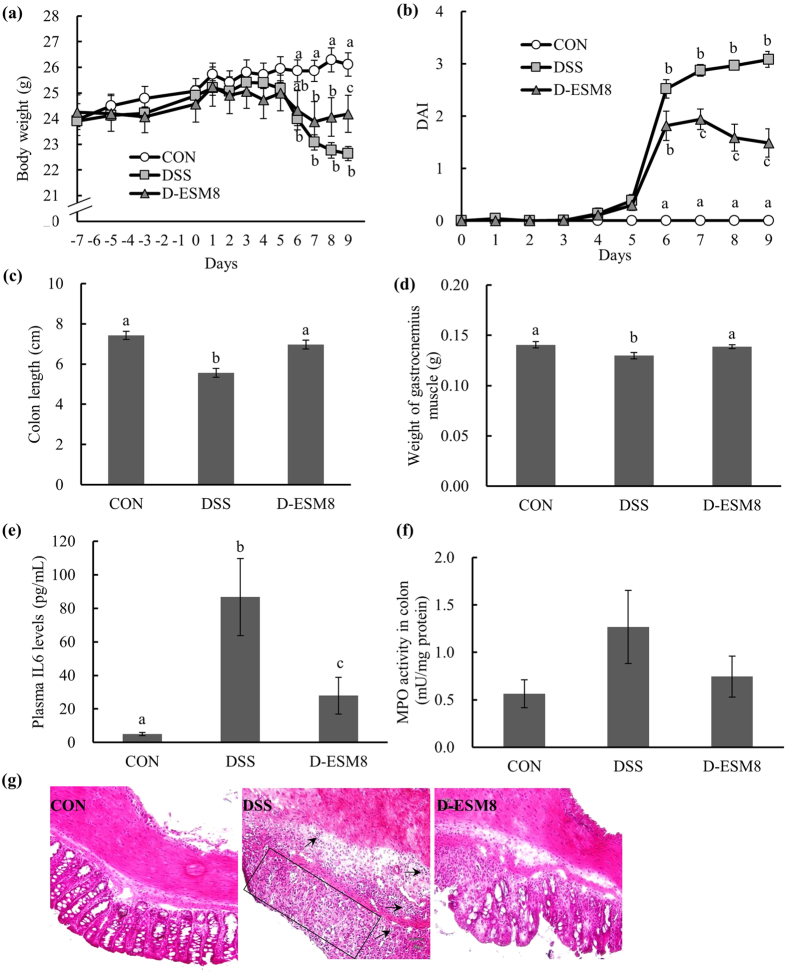
General characteristics of the study groups. (**a**) body weight, (**b**) DAI, (**c**) length of colon, (**d**) weight of the gastrocnemius muscle, (**e**) plasma IL-6 levels, (**f**) MPO activity in colon, and (**g**) H&E staining of colon. The rectangle encircles the part of deprived colon crypts and arrows are pointing to the infiltrated inflammatory cells. CON, control mice; DSS, mice administered only DSS; D-ESM8, mice administered DSS and ESM (80 g kg^−1^). Results are mean ± SE in each group (n = 8). Data with different letters (**a,b,c**) are significantly different at *P* < 0.05 by Dunnett’s test.

The mice that received DSS alone showed significant body weight loss (Fig. 2a) and diarrhea (Suppl. Fig. S2), resulting in significantly severe disease activity index (DAI) values (Fig. 2b) as well as shortened colon lengths (Fig. 2c), reduced weight of the gastrocnemius muscle (Fig. 2d), increased plasma IL-6 levels (Fig. 2e) and augmented colonic MPO activity (*P* = 0.12, Fig. 2f). The D-ESM8 mice revealed low susceptibility to these pathological conditions of IBD, resulting in a significant amelioration of DAI from day 7. In addition, a significant loss of crypts and inflammatory cell infiltration into the mucosa were observed in the H&E-stained colon sections of the DSS group, whereas ESM supplementation revealed clear re-epithelialization of ulcerated areas (Fig. 2g).

### Variations in the colonic gene expression profile

**Figure 3:**
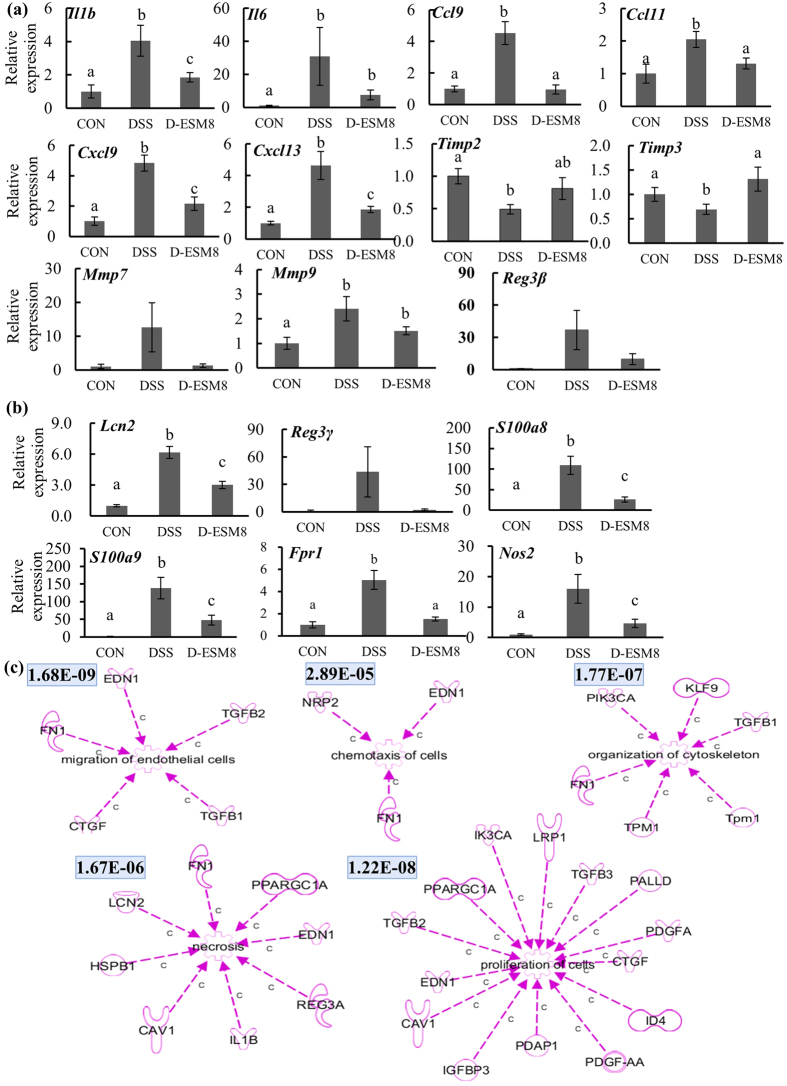
Colonic mRNA expressions of genes related to (**a**) inflammation as evaluated by RT-PCR, (**b**) nutritional immunity by RT-PCR, and (**c**) intestinal cell proliferation and restitution as shown by microarray data. The numbers in the upper left spaces are *P*-values indicating the gene proportion mapping to a function or pathway analyzed via an IPA. V-shape, cytokine/growth factor; spiral, enzyme; dumbbell, transcription regulator; Y-shape, transmembrane receptor; cupule, transporter; three-leaf, kinase; double circle, complex; circle, other; gear, function. CON, control mice; DSS, mice administered only DSS; D-ESM8, mice administered DSS and ESM (80 g kg^−1^). Results are mean ± SE in each group (n = 8). Data with different letters (**a,b,c**) are significantly different at *P* < 0.05 by Dunnett’s test.

To obtain further insight into the modulating effect of ESM during intestinal inflammation, we performed a DNA microarray analysis to unravel the biological pathways involved. The most highly ranked functional clusters and categories for genes that are altered in the colon in accord with the Ingenuity Pathway Analysis (IPA, http://www.ingenuity.com/, date accessed: Jan. 2014) are those involved in the functions of immune cells and epithelial restitution (Suppl. Table S4). In each immune cell function-related category, inflammatory cytokines and chemokines were highlighted, such as *Il1β, Il6*, and members of the chemokine (C-X-C motif) ligand (CXCL) family and the chemokine (C-C motif) ligand (CCL) family. All of these up-regulated genes (*Il1β, Il6, Cxcl9, Cxcl13, Ccl9* and *Ccl11*) in the DSS group were suppressed in the D-ESM8 group (Fig. 3a). Exposure of DSS-treated mice to ESM also down-regulated the gene expression levels of the matrix metalloproteinase (MMP) family (*Mmp7* and *Mmp9*) and up-regulated the gene expression of tissue inhibitors of metalloproteinase (*Timp2* and *Timp3*), which are involved in the breakdown of the extracellular matrix and tissue remodeling in normal physiological processes and disease progression.

In addition, the expression levels of the genes coding for antimicrobial peptides and microbial growth related genes (lipocalin-2, *Lcn2*; regenerating islet-derived 3 gamma, *Reg3γ*; S100 calcium binding protein, *S100a8, S100a9* and formyl peptide receptor 1, *Fpr1*), as well as an oxidative activity-associated gene nitric oxide synthase 2 (*Nos2*) were also lower in the D-ESM8 group compared to the DSS group (Fig. 3b).

Consistent with the ESM’s regulatory action in Caco-2 cells, the ESM diet attenuated the transcription levels of critical intestinal epithelial proliferation- and restitution-related genes, which are involved in the migration of cells, homing, organization of the cytoskeleton, proliferation of cells, and necrosis (Fig. 3c, Suppl. Fig. S3).

### LPS responses in ESM-supplemented mice

**Figure 4:**
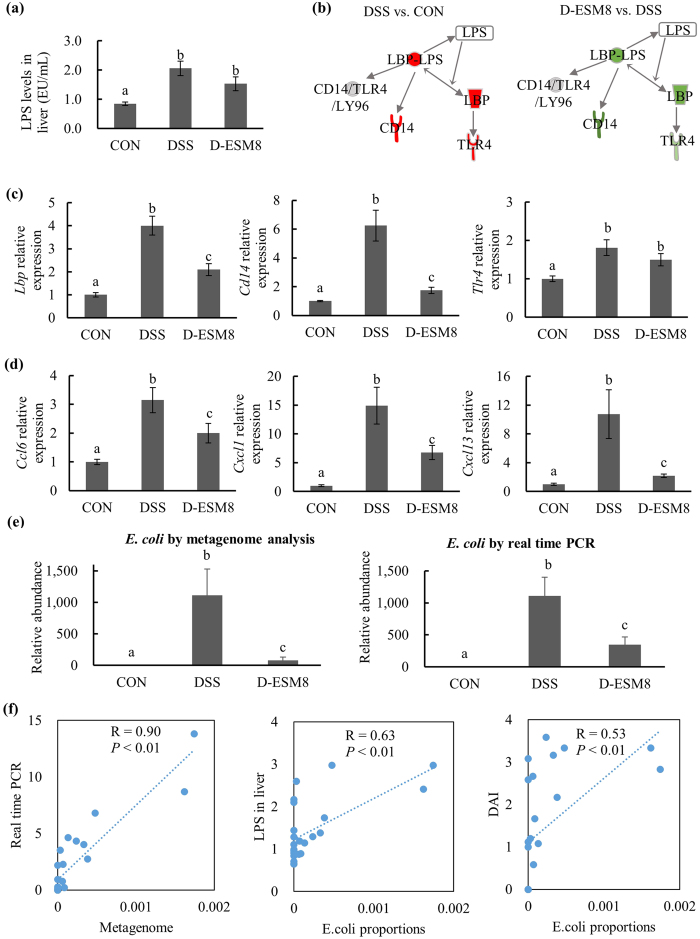
LPS responses in liver and producing bacteria. (**a**) LPS levels in the liver. (**b**) The LPS response-related IPA pathway. Red and green indicate up- and down-regulation, with the more intense color indicating a greater change. (**c**) Hepatic mRNA expressions of genes related to the LPS response and (**d**) inflammation. (**e**) *E. coli* abundance in the cecum content by metagenome analysis and RT-PCR. (**f**) Correlations between *E. coli*, LPS levels and DAI. CON, control mice; DSS, mice administered only DSS; D-ESM8, mice administered DSS and ESM (80 g kg^−1^). Results are mean ± SE in each group (n = 8). Data with different letters (**a,b,c**) are significantly different at *P* < 0.05 by Dunnett’s test.

Since ulcerative colitis is associated with an increased bacterial translocation to the liver and an elevated LPS level is linked to increased intestinal permeability^9^, we examined the LPS level in the liver of DSS-treated mice. This level tended to be suppressed in the D-ESM8 group compared to the DSS group (Fig. 4a). ESM also decreased the hepatic gene expression of biomolecules involved in LPS responses (Fig. 4b), including LPS binding protein (*Lbp), Cd14* and toll-like receptor (*Tlr4)* (Fig. 4c) and the chemokines *Ccl6, Cxcl11* and *Cxcl13* (Fig. 4d). The metagenomic analysis revealed that the relative abundance of one of the LPS-producing pathogenic bacteria, the *Enterobacteriaceae* family member *Escherichia coli (E. coli*) was increased in the DSS groups, whereas ESM restored the *E. coli* abundance to near-normal levels (Fig. 4e).

In addition, there was a remarkably high correlation (*r* = 0.90) between the results of the real-time RT-PCR and the metagenome data of *E. coli* relative abundance (Fig. 4f). Similarly, the positive correlations between the abundance of *E. coli* and the LPS concentration (*r* = 0.63), as well as between the abundance of *E. coli* and the inflammation score DAI (*r* = 0.53) (Fig. 4f) indicate that the suppressive effect of ESM on colitis may be associated with its negative impact on the abundance of *E. coli* which subsequently suppressed inflammation and tissue injury.

### Reduced Th (T helper) 17 number in ESM-supplemented mice

**Figure 5:**
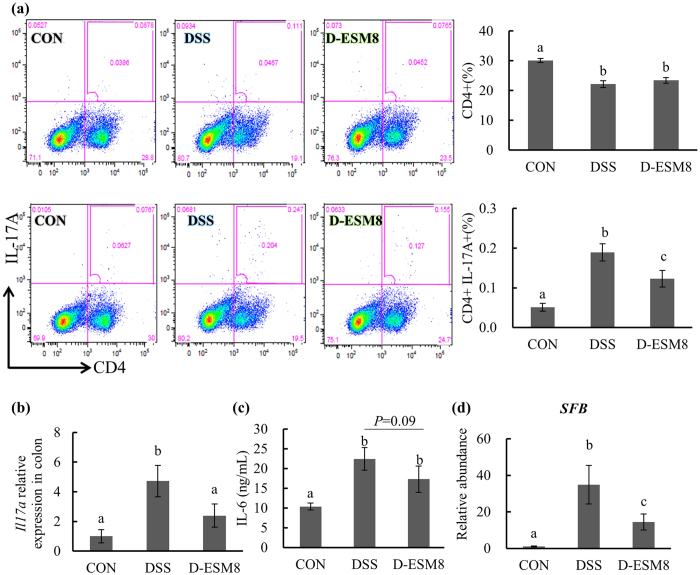
The number of Th17 cells and related bacteria. (**a**) The Th17 number in MLNs shown by the flow cytometric analysis. (**b**) The *Il17a* expression in the colon. (c) The IL-6 levels in the medium of 48 h-incubated MLNs. (**d**) SFB abundance in cecum contents. CON, control mice; DSS, mice administered only DSS; D-ESM8, mice administered DSS and ESM (80 g kg^−1^). Results are mean ± SE in each group (n = 8). Data with different letters (**a,b,c**) are significantly different at *P* < 0.05 by Dunnett’s test.

Given that mesenteric lymph nodes (MLNs) function as the primary gut-associated inductive site where naïve T cells first encounter enteric antigens and are activated as disease-producing colitogenic Th1 and/or Th17 cells^10^,^11^, we isolated MLNs and evaluated the number of Th17 cells by flow cytometry. The results revealed that the DSS administration induced significant CD4^+^ IL-17A frequency, which was significantly attenuated by ESM (Fig. 5a). Corroborating the flow cytometry analysis results, the gene expression findings showed a significantly lower colonic expression of *Il17a* in the D-ESM8 mice compared to that of the DSS mice (Fig. 5b), and there was also a tendency (*P* = 0.09) for low levels of IL-6 in the media of 48 h-incubated MLNs (Fig. 5c). The level of segmented filamentous bacteria (SFB) (Fig. 5d), which promoted the differentiation of Th17 cells, was significantly higher in the DSS group compared to that of the control mice, and this high SFB abundance was reversed in the D-ESM8 group. These findings suggest the possible ability of ESM to regulate the function of SFB and the differentiation of Th17 cells.

### The multifaceted integrated omics analysis revealed ameliorated energy metabolism by ESM supplementation

**Figure 6:**
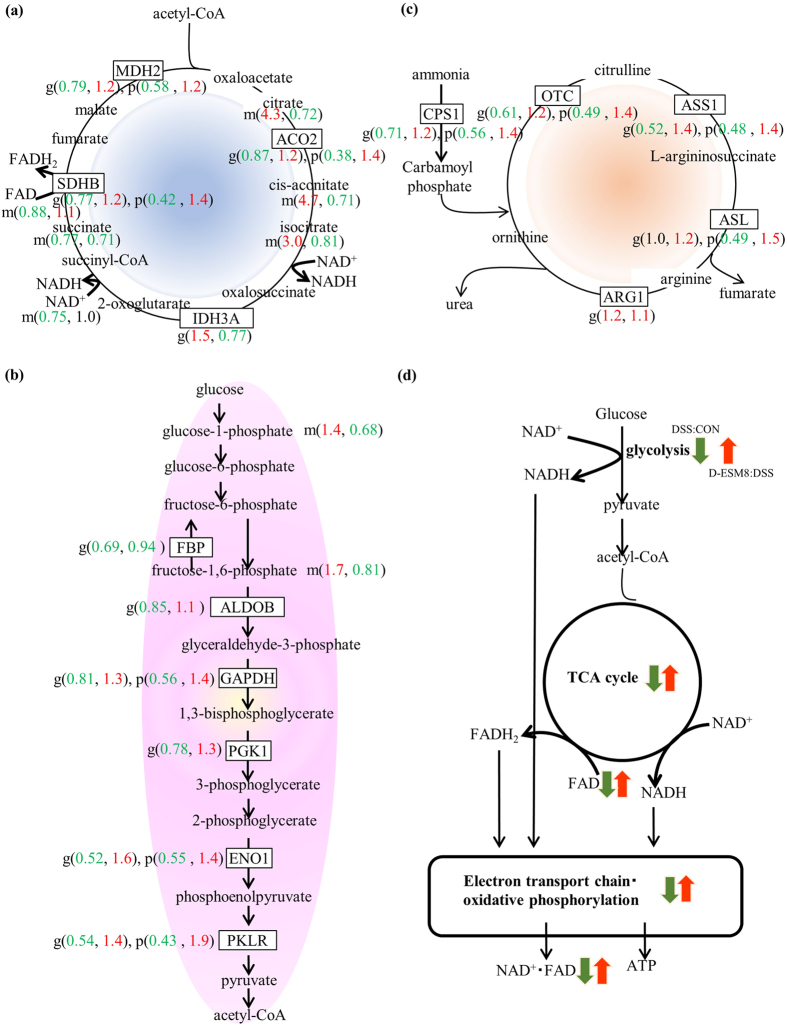
The energy metabolism-related genes, proteins and metabolites in the liver revealed by the omics analysis. (**a**) TCA cycle, (**b**) urea cycle, (**c**) glycolysis pathway. The numbers in () are fold changes of the gene(g) expression, protein(p) and metabolite(m) levels, respectively, where red indicates an increase and green indicates a decrease in the two comparisons (DSS vs. CON and D-ESM8 vs. DSS). (**d**) Correlation diagram of each pathway. The colorful arrows represent promoted or increased (red) and suppressed or decreased (green) in the two comparisons.

Hepatobiliary manifestations are frequently observed in IBD patients, because of the close anatomic link between the gastrointestinal tract and the hepatobiliary system, and due to the mesenteric venous drainage ascending via the portal vein into the liver^9^. This prompted us to conduct an omics analysis to elucidate the relationship between colitis and various hepatic functions. The results of this analysis demonstrated that the top hepatic protein functional categories of the differentially changed proteins were those involved in the urea cycle, gluconeogenesis, glycolysis, the tricarboxylic acid (TCA) cycle, ketogenesis, and fatty acid β-oxidation (Suppl. Table S6). These findings also correspond to the transcriptome profile (Fig. 6); that is, the expressions of these pathway related genes were suppressed in the DSS mice compared to the control mice, whereas the supplementation with ESM elevated these expressions.

Distinct improvements of hepatic intermediates were observed in the TCA cycle (citrate, cis-aconitate, isocitrate) (Fig. 6a, Suppl. Table S8) and glycolysis (glucose-1-phosphate, G-1-P; fructose-1,6-bisphosphate, F-1,6-P) (Fig. 6b) in the DSS mice exposed to ESM. The plasma intermediates of the TCA cycle (citrate, cis-aconitate, isocitrate, 2-oxoglutarate, succinate, fumarate, malate, hydroxyproline and Trp) also indicated that the ESM supplementation improved the weakened glycolysis and the TCA cycle caused by DSS, which is common in inflammatory environments (Suppl. Table S7). Collectively, the expression of energy metabolism-related genes and the levels of proteins and metabolites in glycolysis, the TCA cycle and the urea cycle (Fig. 6a–c) were elevated in the D-ESM8 mice compared to the DSS mice (Fig. 6d).

We also observed changed expressions of proteins involved in mitochondrial dysfunction from functional categories of the proteomic analysis (Suppl. Table S6), including ATP synthase (Atp5c1 and Atp5a1) and NADH hydrolytic enzymes (Ndufab1, Ndufb5, Ndufab10, Ndufv1, Ndufs2, and Ndufs6), which are enzymes involved in the electron transport chain and oxidative phosphorylation (Suppl. Fig. S4). All of these enzymes showed lower levels in the DSS group compared to the CON group, and the levels were preserved in the D-ESM8 group.

### ESM improved microbiota dysbiosis and community structure

As the pathogenesis of IBD results from a pathogenic immune response against the gut microbiota^12^, we next investigated whether the suppressive effects of ESM on DSS-induced colitis also resulted from the alleviation of dysbiosis, by analyzing microbiota functional profiles using a metagenome analysis.

**Figure 7:**
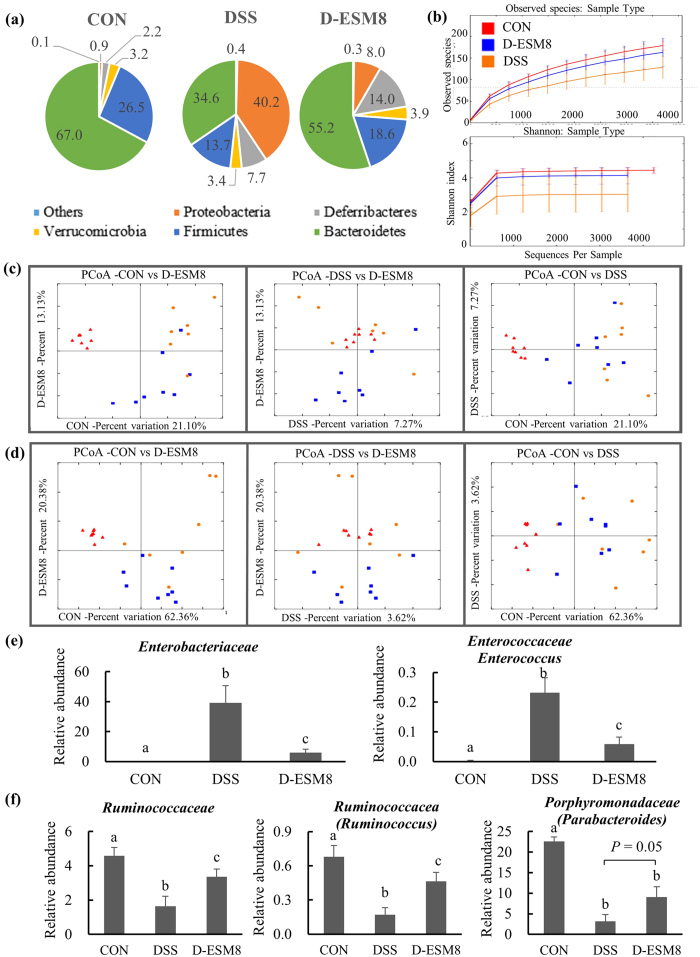
Effects of ESM on microbiota dysbiosis and structure as revealed by the metagenome analysis. (**a**) Phylum level of microbiota profiling (%). (**b**) Rarefaction curves. (**c**) Unweighted UniFrac-based principal coordinate analysis. (**d**) Weighted UniFrac-based principal coordinate analysis. (**e**) Abundance of IBD-related bacteria. (**f**) SCFA-producing bacteria. Group names are as in the earlier figures. Results are mean ± SE in each group (n = 8). Data with different letters (**a,b,c**) are significantly different at *P* < 0.05 by Dunnett’s test.

The analysis at the phylum level revealed that the relative abundance of the three most prevalent bacterial phyla was significantly different among the three experimental groups. *Bacteroidetes* decreased from 67.0% in CON to 34.6% in DSS and recovered to 55.2% in ESM; *Firmicutes*, from 26.5% to 13.7% and to 18.6%; *Proteobacteria*, from 0.9% to 40% and to 8% (Fig. 7a), respectively.

We then assessed two classifications of bacterial diversity termed α and β. According to the α-diversity (rarefaction plot) analysis using Observed species and the Shannon index, the DSS mice exhibited the lowest species richness among the three groups (Fig. 7b), which is expected since a lower richness of bacterial species has been associated with disease states, including IBD in humans and DSS colitis in mice^13^. The higher richness of the ESM group matched the beneficial effects of ESM on colitis.

We also analyzed the β-diversity — which reveals distinctive views of community structure — by using two phylogenetic distance metrics, i.e., weighted and unweighted Unifrac distances, which measure the presence and absence of abundant lineages and particular bacteria taxa, respectively. The percent variations by the unweighted metric of the CON, DSS and D-ESM8 groups were 21.10%, 7.27% and 13.13%, respectively (Fig. 7c), which indicates that rare bacteria altered the community structure of these groups.

The clustering of the CON, DSS and D-ESM8 groups by the weighted UniFrac distances showed 62.36%, 3.23% and 20.38% variations, respectively indicating that bacterial communities were more evident (Fig. 7d). Regardless of the rare and abundant bacteria, these data suggest that microbial communities are differentially improved by ESM, indicating that the changes in microbiome diversity may be related to the anti-inflammatory effects of ESM.

Increased *Enterobacteriaceae*, which is a marker of intestinal inflammation and oxidative stress in human IBD and murine colitis, was detected significantly more frequently in the inflammatory DSS groups (*Proteobacteria* phylum, reaching 39% of the total bacteria) compared to the CON group (Suppl. Table S9). Conversely, *Enterobacteriaceae* were detected significantly less frequently in the D-ESM8 group (approx. 6%). Similarly, the DSS administration increased the numbers of *Enterococcaceae*, which were counteracted to near-normal levels by ESM supplementation (Fig. 7e).

In addition, considering that the metabolites of gut microbiota such as short-chain fatty acids (SCFAs) are the primary energy source for colonocytes and directly influence host gene expression, we further focused on the presence of SCFA-producing microbiota (Fig. 7f). The DSS group had a reduced abundance of the SCFA producers *Ruminococcaceae (Firmicutes* phylum, comprising approx. 10% of the total bacteria) and *Porphyromonadaceae (Bacteroidetes* phylum) known to be decreased in IBD, all of which were restored by ESM.

## Discussion

The intestinal epithelium is both a highly efficient absorptive and residual surface for nutrients and fluids and a tight barrier between the intestinal bacteria and the body’s immune system^14^. An impairment of the integrity of the intestinal epithelium may subsequently increase the risk of intestinal inflammation and cancer^15^. The processes of epithelial restitution involve cell migration, homing, cytoskeleton organization, cell proliferation and more, which are mainly activated by growth factors.

Members of the transforming growth factor (TGF) family are multifunctional and play central roles in the induction of intestinal epithelium repair after mucosal injury^16^. Importantly, TGFβ2 and TGFβ3 regulate cell proliferation, growth, differentiation and migration^16^. Along with the promoted proliferation of ESM-supplemented Caco-2 cells *in vitro*, we noted the factors responsible for restoring the impaired epithelial integrity in DSS-induced colitis in the microarray data. Coupled with the RT-PCR validation, these results revealed that ESM ameliorated the low gene expression of growth factors and metalloproteinase and that it induced epithelial restitution. More specifically, our data demonstrate that ESM may potentially promote intestinal restitution, as it can strongly limit inflammation, restore microbial diversity, and heal ulcerated lesions in IBD.

Since no similar effect was observed from hydrolyzed eggshell membrane (data not shown), the epithelial restitution of ESM might be attributed to ESM collagen or its degradation dipeptides such as prolyl-hydroxyproline, which are known to promote the proliferation of fibroblast-like cells^17^.

Inflammatory responses in the colon are accompanied by a high demand for energy production, which is used by immune cells to recruit inflammatory lesions and by epithelial cells to fuel cell repair and regeneration in the tissues^18^,^19^. Since elevated protein catabolism in inflammatory cells also increases the energy demand, the levels of amino acids and TCA intermediates and the expression of urea cycle enzymes are reduced in the serum and colonic mucosal tissue of IBD patients^20^,^21^. Intestinal mucosal inflammation and diarrhea, which can limit the absorption of food-derived proteins, are also probable reasons for the decrease in blood amino acid concentrations. In our present results, the significantly high gastrocnemius muscle weights in the D-ESM8 mice indicate a lower energy demand than that in the DSS group, thereby inhibiting the degradation of muscle tissue, which was also reflected by high levels of plasma amino acids and the recovery of the TCA-cycle intermediates in the D-ESM8 group.

Consistent with the proteome results, our metabolome analysis revealed high TCA cycle and glycolysis intermediates in the livers of the DSS mice, indicating a hepatic accumulation of these intermediates. The decreased concentrations of plasma TCA-cycle intermediates in the DSS group also reflect the stationary state of glycolysis and the TCA cycle in the liver. However, in the D-ESM8 group, the hepatic concentrations of TCA-cycle and glycolysis intermediates from both the metabolic and proteomics analyses are contrary to those of the DSS group. This indicates an enhanced TCA cycle and glycolysis, which may be due to an efficient energy supply in the D-ESM8 group, resulting in the reduction of weight loss.

The upregulated expression of urea-cycle proteins in the D-ESM8 group suggests improved nitrogen balance (which is often impaired in IBD) and less inflammatory acute-phase protein synthesis, since there is a progressive downregulation of urea-cycle gene mRNAs and an opposite upregulation of acute-phase protein mRNAs^22^, as also shown by the expression of LPS-responsive genes and chemokines. The similar changes of genes involved in glycolysis and the TCA and urea cycles corresponded to the results of the transcriptome analysis. These results provide novel information about IBD, which is helpful for studying the pathogenesis mechanisms of the disease as well as finding rational therapeutic approaches for the treatment of IBD.

In addition, the up-regulated expression of proteins involved in the mitochondrial electron transport chain and oxidative phosphorylation observed in the DSS group in response to ESM treatment indicate improvement in mitochondrial dysfunction, including a low electron transfer system, low oxidative phosphorylation, impaired ATP synthesis and decreased NAD^+^ production. Moreover, the metabolome profiles indicate a significantly higher flavin adenine dinucleotide (FAD) level in the D-ESM8 group, supporting the recovery of the electron transport chain by dietary ESM. These results indicate that the increased energy production in the D-ESM8 mice from accelerated glycolysis and the TCA cycle via the enhanced electron transport chain and oxidative phosphorylation may have led to the reduction of the weight loss and the improved disease symptoms.

As commensal bacteria contribute to immune system development, the differentiation of regulatory T cells (Tregs) and Th17 cells, the pro- and anti-inflammatory cytokine balance and intestinal barrier functions^23^, we also investigated the relationship between inflammation and gut microbial function. Our analysis of the cecal microbiota composition revealed significant changes in potent inducers of Th17 and Tregs. The aberrant expansion of SFB (which are Gram-positive, spore-forming obligate anaerobes) in DSS group of our study is consistent with a role for *in vivo* pathogenic Th17 induction^24^. The suppressed overgrowth of SFB and restrained Th17 cell responses by the ESM supplementation indicate the role of ESM in bacterial metabolism and the intestinal homeostasis of Th17 cells in the DSS-induced mice model of IBD.

SCFAs have been shown to regulate the homeostasis of colonic Tregs by inducing the differentiation of Tregs from naïve T cells via an enhancement of histone H3 acetylation in the forkhead box protein 3 (Foxp3) locus^25^. In our study, the low abundance of SCFA producers in the DSS group also indicates an imbalance in energy metabolism, which is reflected by the metabolome and proteome data. The increased SCFA producers *Ruminococcaceae* and *Porphyromonadaceae* in the D-ESM8 group may promote the enteric fermentation of SCFAs by releasing ammonia or amine from indigestible ESM, thus improving the ratio of carbohydrate:nitrogen as fermentative substrates, affecting host inflammation via increased SCFA availability and resulting in the accelerated energy metabolism in mice. These observations also highlight the idea that ESM might cross-regulate commensal microbiota and the mucosal immune system to maintain intestinal homeostasis.

LPS is produced by Gram-negative bacteria such as *E. coli* that exhibit pathogen-like behaviors and cause inflammatory destruction of the intestinal epithelial barrier. In our metagenome data, *E. coli* was particularly highly enriched in the DSS group, which may be associated with diarrhea and tissue damage due to toxin secretion or stimulated cytokine production^26^. The decreased LPS level and LPS immune reaction in the livers of the DSS mice in response to ESM treatment indicate that ESM is associated with a restored ability to detoxify bacterial LPS, thereby regulating dysbiosis in IBD pathogenesis.

Under inflammatory settings, there is a defense mechanism known as ‘nutritional immunity’ which can influence bacterial growth by releasing antimicrobial protein and changing the nutritional environment^27^. In our study, the gene expression of some of the antimicrobial peptides which are known to be bactericidal was attenuated in the DSS group by ESM treatment. For instance, the release of the C-type lectin Reg3γ contributes to the luminal clearance of the opportunistic pathogen *Enterococcus*, thereby playing a role in host defense and protecting the mucosa from injury.

Inflammatory responses in the colon begin with an infiltration of neutrophils and macrophages^28^, and the activated macrophages produce a number of potent inflammatory cytokines. The transepithelial migration of neutrophils and the subsequent release of calprotectin, a heterodimer composed of S100A8 and S100A9, from dead neutrophils reduce the availability of zinc, which is required for bacterial growth in the intestinal lumen^27^. Notably, neutrophil migration to the intestinal lumen results in the generation of organized intraluminal structures that encapsulate commensals and limit their contact with the epithelium, the process of which is regulated by the high-affinity *N*-formyl peptide receptor, Fpr1^29^. Additionally, Lcn-2 prevents bacterial iron acquisition by binding and sequestering enterobactin^27^,^30^, and then it inhibits the growth of bacteria.

In colonic inflammation, the oxidation by-products of the inflammatory host response such as nitrate and *N*-oxides generated by inducible nitric oxide synthase (iNOS) can serve as exogenous electron acceptors for anaerobic respiration and modify the bacterial growth conditions to a large extent. These conditions are thus predicted to favor the growth of *Enterobacteriaceae*, because members of this family are more likely to encode the enzymes required for nitrate respiration.

The luminal growth of *E. coli* is likely further fueled by the respiration of other inflammation-derived electron acceptors^27^. The markedly up-regulated expression of *Nos2* and the increased abundance of *E. coli* in the present DSS group support the generation of exogenous electron acceptors by the inflammatory host response, resulting in uncontrolled expansion of *Enterobacteriaceae,* and leading to intestinal dysbiosis. Taking the significantly lower expression of cytokines and chemokines and *S100A8, S100A9, Fpr1, Nos2* and *Lcn2*, and lesser abundance of *Enterobacteriaceae* and *E. coli* as well as lower activity of MPO (which is the marker of neutrophil migration) in the ESM group compared to the DSS group into consideration, we speculate that ESM may modulate a barrier-protective host response. Our findings further indicate that the intestinal microflora and innate immune system respond to both ESM and low inflammatory host response nutritional immunity, thereby imposing low forces on microbial growth and restored microbial diversity.

With respect to these impacts of ESM on microbiota, we have developed the following hypothesis. Since simple carbohydrates and proteins are digested and absorbed in the upper gastrointestinal tract, complex carbohydrates (e.g., fiber or mucus carbohydrates) or non-digestible proteins (e.g., gluten) are the main nutrients supporting the growth of obligate anaerobic bacteria in the colon. It appears that ESM (which can provide more non-digestible proteins) may have a physiological function similar to that of dietary fiber to stimulate cecal fermentation by providing nitrogen sources to intestinal bacteria. Simultaneously, bacteria that are able to utilize amino acid metabolism dominate the energy supply as a long-term effect in inflamed tissue. Further studies, particularly regarding the mechanisms responsible for restoring a balanced community structure, are required to define potential targets for intervention strategies to improve colitis.

## Conclusion

**Figure 8:**
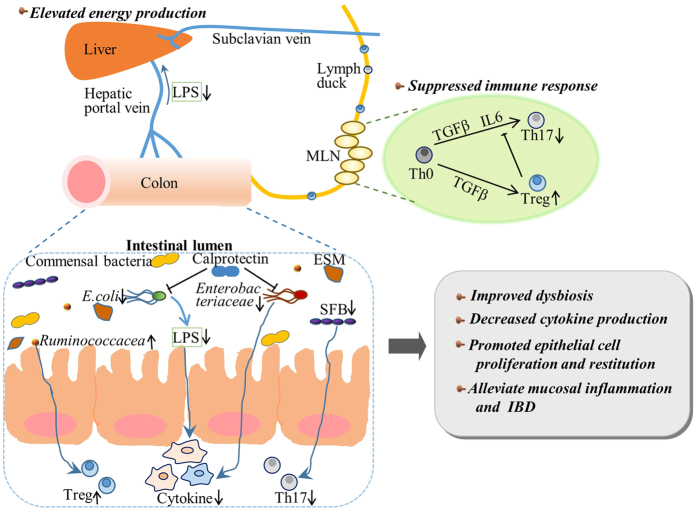
Schematic representation of the mechanism underlying the suppressing effects of ESM against the development of colitis. The genes in light green ovals had a strong tendency for improvement, and the genes in dark green or dark red ovals showed a significant improvement following the ESM supplementation.

The results of the present study including those of our multifaceted integrated omics analyses provide new insight into ESM as a novel and effective intervention in optimal IBD management, particularly with regard to reducing the severity of colitis and preventing dysbiosis, which is characterized by an increased diversity of bacteria and decreased absolute numbers of pathogenic bacteria such as *Enterobacteriaceae* and *E. coli*. In addition, the promoted epithelial cell growth and restitution contribute to low antigen influx and suppressed immune response by down-regulating chemokine expression, accompanied by a low number of Th17 cells and elevated energy production. Together our present findings increase our comprehensive understanding of the interactions between the host and its resident microbiota and their respective roles in IBD (Fig. 8). Notably, taking into consideration the minimal side effects of ESM as a by-product in the manufacturing of egg products, the use of this natural, low-priced waste product appears to be a promising candidate for the prevention and treatment of IBD, leading to a reduction of healthcare costs and a lessened environmental load, which are the most intriguing and attractive implications of this study.

## Materials and Methods

### Culture of Caco-2 cells and LPS stimulation

Cells of the human colon epithelial cancer cell line Caco-2 (the American Type Culture Collection, Rockville, MD, USA) were cultured in a culture medium composed of Dulbecco’s modified Eagle’s medium (Gibco, Grand Island, NY, USA) with 10% fetal bovine serum (FBS, Sigma-Aldrich, St. Louis, MO), 1% non-essential amino acids (Gibco), 100 U/mL penicillin and 100 μg/mL streptomycin (Sigma-Aldrich) at 37 °C in a 5% CO_2_ environment.

Cells were seeded into six-well tissue culture plates (Corning, NY, USA) (1 × 10^6^ cells/well) in 2 mL of culture medium and incubated for 14 days. Next, the cells were further conditioned in the medium supplemented with 1 and 2 mg/mL of ESM (ALMADO, Tokyo, Japan) fine powder for 24 h, followed by treatment with 1 mg/mL of LPS (0111: B4, Sigma-Aldrich) for 24 h. The medium was then collected for the lactate dehydrogenase (LDH) assay and cytokine IL-6 measurement (High-Sensitivity Human ELISA kit, Abcam, Cambridge, MA, USA), and the cells were suspended in 2.5 mM HEPES (Nacalai Tesque, Kyoto, Japan) containing 1% Tween for RNA extraction. Cell viability was evaluated by WST-8 assay using the Cell Counting Kit-8 (Dojindo, Mashiki, Japan) according to the manufacturer’s manual. The cell viability is presented as the fold-change values of the ESM group to the CON group, the value of which was considered as 1. All methods were performed in accordance with relevant guidelines and regulations.

### Animals and induction of colitis

Seven-week-old male C57BL6/J mice were obtained from Charles River Japan (Charles River Laboratories Japan, Yokohama, Japan) and housed individually under controlled conditions (50 ± 10% relative humidity, 23 ± 2 °C, and a 12/12-h light/dark cycle). After 3 days of acclimatization, the mice were divided into three groups (n = 8 per group) with equal mean body weights. They were fed either the control AIN-93G basal diet (Oriental Yeast Co., Tokyo; Suppl. Table S1) (DSS group,) or the 8% ESM fine powder-supplemented diet (Suppl. Table S1) (D-ESM8 group) for 7 days, after which colitis was induced by administering drinking water that contained 1.5% (w/v) DSS (molecular weight, 40 KDa; MP Biomedicals, Irvine, CA, USA) for 9 days while the same experimental diets were provided. The 8% ESM diet was adjusted with cornstarch and casein using 46% of the digestibility of ESM to maintain the caloric and protein balance (Suppl. Table S1). Normal control mice (CON group) were given the AIN-93G basal diet and drinking water without DSS for the entire course of the experiment.

The DAI was monitored every day after the start of the DSS-water administration using three scores for body weight, stool consistency, and fecal blood of the mice, then calculated by averaging these three scores^31^. All animal experiments were approved by The University of Tokyo Animal Care and Use Committee (no. P13-739), and the animals were maintained with humane care per the Committee’s requirements.

### Blood collection and tissue harvesting

At the time of sacrifice, all mice were deeply euthanized with sodium pentobarbital followed by bleeding from the carotid artery. Plasma was obtained by centrifuging blood immediately at 1000× g for 15 min at 4 °C. The distance between the ileocecal junction and the proximal rectum was measured as the colon length. The excised colon, liver, cecal contents, gastrocnemius muscle and mesenteric adipose tissues were snap-frozen in liquid nitrogen and stored at −80 °C until their use in assays.

### Biochemical assays

The concentration of IL-6 in cell culture media and plasma, and the colonic myeloperoxidase (MPO) activity were measured by using an enzyme-linked immunosorbent assay (ELISA) kit (Thermo Scientific, Rockford, IL) and a colorimetric kit (BioVision, Palo Alto, CA, USA), respectively according to the manufacturers’ instructions. In order to prepare the MPO samples, the colon tissues were homogenized in 10-fold MPO assay buffer and centrifuged at 13,000× g at 4 °C for 10 min, and the supernatants were used for the determination of enzymatic activity. The protein content was assayed by a Bradford assay (Bio-Rad, Hercules, CA, USA), and the results are expressed as activity units per mg protein.

### Colon histology

Colon portions were embedded in OCT (optimal cutting temperature) compound (Sakura Finetek, Torrance, CA, USA) and snap-frozen next in liquid nitrogen. The tissues were sectioned at 5 μm with a cryostat (CryoStar NX70; Thermo Scientific, Waltham, MA, USA). Hematoxylin and eosin (H&E) staining was then conducted, and we scanned the sections at 200× using a polarizing microscope (BX51, Olympus Optical, Tokyo, Japan).

### Isolation of MLNs and the flow cytometric analysis

MLNs were extirpated and incubated in 1.0 mg/mL of collagenase (Wako, Tokyo, Japan) and 10 μg/mL DNase (Roche, Mannheim, Germany) for 1 h and filtered using 40-μm filters (BD Falcon, San Jose, CA, USA) to collect the single cells. The cells were washed and resuspended in complete medium (RPMI 1640 medium containing 10% FBS, 100 U/mL penicillin, and 100 μg/mL streptomycin).

Next, 2 × 10^6^ MLNs cells were transferred to a 96-well plate and incubated at 37 °C for 7 h in complete medium containing anti-mouse CD28 antibody (2 μg/mL) on anti-mouse CD3 antibody-coated wells, followed by the addition of monensin (0.1 μg/well) for 4 h before the end of the incubation period. Prior to staining, Fc-receptors were blocked using CD16/Cd32 to prevent nonspecific binding of the antibody. The cells were stained for extracellular CD4 using FITC-conjugated anti-mouse CD4 antibody. Subsequently, the cells were fixed and permeabilized using 0.5% saponin buffer and then stained for intracellular IL-17A using Alexa Fluor 647 anti-mouse IL-17A antibody. The flow cytometric analysis was conducted using FACSverse (BD Biosciences, San Jose, CA, USA) followed by the analysis using FlowJo software (Tree Star, Ashland, OR, USA). All of the antibodies and reagents used were purchased from BD Pharmingen (San Diego, CA, USA).

### The total RNA extraction protocol and the real-time RT-PCR

We used TRIzol reagent (Invitrogen, Carlsbad, CA, USA) and an RNA Isolation Kit (NucleoSpin^®^ RNA Π, Macherey-Nagel, Düren, Germany) to extract the harvested cells’ total RNA and the total RNA of the frozen liver and colon, respectively. The primer sequences and conditions for our real-time reverse transcription-polymerase chain reaction (RT-PCR) are given in Suppl. Table S2 and Supplemental Methods. We normalized the expression values for the specific genes against the expression levels of glyceraldehyde-3-phosphate dehydrogenase (GAPDH), β-actin or 60S acidic ribosomal protein P1 (Rplp1) in the cells, liver or colon, respectively. The data are presented as the fold-change values of the normalized mRNA amounts compared to the values of the control mice.

### Transcriptome analysis and ingenuity pathway analysis (IPA)

The microarray analysis was carried out using pooled total hepatic and colonic RNA from each respective group as described^32^. For the genome-wide expression profiles, we used Mouse Genome 430 2.0 Array GeneChips (Affymetrix, Santa Clara, CA, USA). We analyzed the scanned images by using Affymetrix Microarray Suite ver. 5.0 software to determine the gene expression ratios for the CON versus DSS mice and for the DSS versus D-ESM8 mice. We defined ‘differentially expressed’ genes as those with expression ratios >1.5-fold. We then mapped these genes by using the IPA’s Pathway Explorer function.

### Proteome analysis protocol and the identification of regulated proteins

The hepatic proteome analysis was conducted as described^8^ in Supplemental Methods. A cut-off for *p*-values < 1.0 and confidence thresholds >1.25 were included for the identification of unique proteins using the IPA classification system.

### Metabolome analysis

The liver and plasma samples were measured by a capillary electrophoresis-time of flight mass spectrometry (CE-TOFMS) analysis (Agilent, Palo Alto, CA, USA)^31^ in Supplemental Methods.

### Cecal bacterial DNA extraction and metagenome analysis

Metagenomic DNA was extracted from cecal contents using an QIAamp Stool Mini Kit (Qiagen, Hilden, Germany) following the manufacturer’s guidelines. To amplify the variable regions 3 and 4 of the 16S rRNA gene, we used the primers (5′-CCTACGGGNGGCWGCAG-3′ and 5′-GACTACHVGGGTATCTAATCC-3′), and modified to contain Illumina adapters and barcode sequences (Supplemental Methods) to allow for sequencing. We conducted a library size and quantification analysis with the Agilent 2100 Bioanalyzer (Agilent Technologies, Santa Clara, CA, USA) and pooled all the libraries on a single Illumina MiSeq run (MiSeq Reagent Kit V3, 600 cycles, Illumina, San Diego, CA, USA) according to the manufacturer’s specifications in order to generate paired-end reads of 300 bases in length in each direction.

The overlapping paired-end reads were merged by fastq-join using Quantitative Insights Into Microbial Ecology (QIIME v1.8.3) software (http://www.qiime.org). The chimeric sequences were removed using the USEARCH method against the Greengenes alignment (v gg_13_8) following which the reads longer than 250 bp and an average quality score above 30 were retained.

The resulting sequences were assigned to operational taxonomic units (OTUs) with a threshold of 97% pair-wise identity, and representative sequences were classified taxonomies using the Ribosomal Database Project (RDP) classifier in QIIME according to the Greengenes OTU database. Distances between samples were visualized graphically using Principal Coordinate Analysis (PCoA) of the weighted and unweighted pair group method with arithmetic mean (UPGMA) clustering. Differences of taxa were analyzed using the non-parametric Kruskal-Wallis test, the results of which were corrected for multiple comparisons using the Benjamin-Hochberg false discovery rate (FDR) adjustment. FDR values < 0.05 were considered significant. Quantitative real-time PCR was performed for the detection of SFB and the validation of several pathogenic bacteria; the specific primers are shown in Suppl. Table S2.

### Statistical analysis

The cell culture and animal experiment results are presented as the mean value ± standard error (SE) of the mean. We performed a one-way ANOVA (analysis of variance) and Dunnett’s test to evaluate the differences between the data means. As non-parametric methods, we used Kruskal-Wallis and Steel’s tests to evaluate the DAI values. *P-*values < 0.05 were accepted as significant.

## Additional Information

**How to cite this article:** Jia, H. *et al*. Eggshell membrane powder ameliorates intestinal inflammation by facilitating the restitution of epithelial injury and alleviating microbial dysbiosis. *Sci. Rep.* **7**, 43993; doi: 10.1038/srep43993 (2017).

**Publisher's note:** Springer Nature remains neutral with regard to jurisdictional claims in published maps and institutional affiliations.

## Supplementary Material

Supplementary Information
