# Supplementary material for: Eggshell membrane powder ameliorates intestinal inflammation by facilitating the restitution of epithelial injury and alleviating microbial dysbiosis
Source: Sci Rep. 2017 Mar 8;7:43993. doi: 10.1038/srep43993 (PMC5341015; doi:10.1038/srep43993)
Supplement: Supplementary Information [file srep43993-s1.pdf]

Supplementary materials

Eggshell membrane powder ameliorates intestinal inflammation by facilitating the restitution of epithelial injury and alleviating microbial dysbiosis

Huijuan Jia[1,*], Manaka Hanate[2], Wanping Aw[1,3], Hideomi Itoh[4], Kenji Saito[1], Shoko Kobayashi[5], Satoshi Hachimura[6], Shinji Fukuda[3], Masaru Tomita[3], Yukio Hasebe[7] and Hisanori Kato[1,2,*]

Abbreviations:

Acly, ATP citrate lyase
ACO2, aconitase 2, mitochondrial
ALDOB, aldolase B, fructose-bisphosphate
ARG1, arginase 1
ASL, argininosuccinate lyase
ASS1, argininosuccinate synthase
Atp5a1, ATP synthase, H+ transporting, mitochondrial F1 complex, alpha subunit 1, cardiac muscle
Atp5c1, ATP synthase, H+ transporting, mitochondtial F1 complex, gamma polypeptide 1
Cav1, caveolin 1
Ccl, chemokine (C-C motif) ligand
Cd14, CD14 antigen
Cfh, complement component factor h
Col18a1, collagen, type XVIII, alpha 1
CPS1, carbamoyl phosphate synthetase 1
Ctgf, connective tissue growth factor
Cxcl, chemokine (C-X-C motif) ligand
DAI, disease activity index
DSS, dextran sulfate sodium
ESM, Eggshell membrane
EDN1, endothelin 1
E. coli, *Escherichia coli*
Elovl6, ELOVL family member 6, elongation of long chain fatty acids
ENO1, enolase 1, alpha non-neuron:
F-1,6-P, fructose-1,6-bisphosphate
FAD, flavin adenine dinucleotide
Fasn, fatty acid synthase
FBP1, fructose bisphosphatase 1
Fn1, fibronectin 1
Fpr1, formyl peptide receptor 1
G1P, glucose-1-phosphate

GAPDH, glyceraldehyde-3-phosphate dehydrogenase:
H&E, haematoxylin and eosin
HDL, high-density lipoprotein cholesterol
Hspb1, heat shock protein 1
IBD, inflammatory bowel disease
Id4, inhibitor of DNA binding 4
IDH3A, isocitrate dehydrogenase 3 (NAD+) alpha
IGFBP3, insulin-like growth factor binding protein 3
IL, interleukin
iNOS, inducible nitric oxide synthase
IPA, ingenuity pathway analysis
iTRAQ, isobaric tag for relative and absolute quantitation
Klf9, kruppel-like factor 9
LBP, LPS binding protein
Lcn2, lipocalin-2
LDH, lactate dehydrogenase
LPS, lipopolysaccharide
MDH2, malate dehydrogenase 2, NAD (mitochondrial)
Me1, malic enzyme 1
MLNs, mesenteric lymph nodes
MMP, matrix metallopeptidase
NAD+, nicotinamide adenine dinucleotide
Ndufab, NADH dehydrogenase (ubiquinone) 1, alpha/beta subcomplex
Ndufb5, NADH dehydrogenase (ubiquinone) 1 beta subcomplex, 5
Ndufs, NADH dehydrogenase (ubiquinone) Fe-S protein
Ndufv1, NADH dehydrogenase (ubiquinone) flavoprotein 1
NEFA, non-esterified fatty acid
Nos2, nitric oxide synthase 2, inducible
OTC, ornithine transcarbamylase
OTU, operational taxonomic unit
Palld, palladin, cytoskeletal associated protein
PCoA, Principal Coordinate Analysis
PDGFα, platelet-derived growth factor, alpha
PGK1, phosphoglycerate kinase 1
Pik3ca, phosphatidylinositol 3-kinase, catalytic, alpha polypeptide
PKLR, pyruvate kinase liver and red blood cell
Ppargc1a, peroxisome proliferative activated receptor, gamma, coactivator 1 alpha
QIIME, Quantitative Insights Into Microbial Ecology
Reg3β, regenerating islet-derived 3 beta
Reg3γ, regenerating islet-derived 3 gamma

S100a8, S100 calcium binding protein A8
S100a9, S100 calcium binding protein A9
Scd1, stearoyl-CoA desaturase-1
SCFAs, short-chain fatty acids
SDHB, succinate dehydrogenase complex, subunit B, iron sulfur (Ip)
SFB, segmented filamentous bacteria
TC, total cholesterol
TCA, tricarboxylic acid
Th, T helper
TG, triglyceride
TGFβ, transforming growth factor-beta
TIMP, tissue inhibitor of metalloproteinase
TLR4, toll-like receptor
TPM2, tropomyosin 2
Tregs, regulatory T cells

Methods

Cell cytotoxicity assay

Cytotoxicity was assessed by a lactate dehydrogenase (LDH)-Cytotoxic Test kit (Wako, Tokyo). LDH was measured according to the manufacturer's instructions and cytotoxicity was determined as followed; cytotoxicity (%) = (LDH in the culture medium) / (LDH in the culture medium + LDH in the cell).

Cell viability assay

Cell viability was evaluated by WST-8 assay (Cell Counting Kit, Dojindo). A density of 5×10^3 cells/well were seeded into 96 well plates in 100 □L culture medium and incubated for 2 days. Cells were further treated with the medium including ESM (1, 2, 3 mg/mL) for 24 hours, followed by treatment with 1 mg/mL LPS for 24 hours. Then the medium was replaced with the medium added with 10% WST-8 solution and incubated for 2 hours. Cell viability was determined by colorimetric comparison at an absorption wavelength of 450 nm and 650 nm; Cell viability = A450 – A650.

Biochemical assays

The hepatic levels of triglyceride (TG), total cholesterol (TC), high-density lipoprotein cholesterol (HDL) and non-esterified fatty acid (NEFA) were measured enzymatically using commercial kits (Wako, Tokyo) after extraction from the frozen livers.

RT-PCR conditions

RT-PCR was performed using SYBR Green EX (Takara Bio, Madison, WI, USA) on the real-time PCR detection system (Takara Bio, Shiga, Japan). Final mixture (12.5μl) for RT-PCR consisted of 1× SYBR Premix Ex Taq Mix (Takara), 0.4 μM of primers (forward and reverse) and 1 μL cDNA. The PCR program

was initiated by a 10 min at 95 °C followed by 30 thermal cycles, each of 10 s at 95 °C, 5 s at 55 °C, and 30 s at 70 °C.

Proteome analysis

Briefly, pooled protein (100 µg) were used for cysteine blocking, digested, and labeled with the isobaric tag for relative and absolute quantitation (iTRAQ) tags according to the manual of the 4-plex iTRAQ labeling kit (AB SCIEX, Framingham, MA, USA). The reconstituted samples (50 µL) were subjected for NanoLC-MS/MS analysis (AB SCIEX). Protein identification and quantification for iTRAQ samples were conducted using ProteinPilot software (ver. 4.0, AB SCIEX, Framingham, MA) with 95% confidence.

Metabolome analysis

Briefly, frozen samples (n=8 in each group) were mixed with 1500 µL of 50% acetonitrile in water (v/v) and homogenized by a multi-sample homogenizer (Shake Master Neo, Bio Medical Science, Tokyo, Japan), then centrifuged at 2,300×g at 4°C for 5 min. The supernatant was filtrated through 5 kDa cut-off filters (Ultrafree-MC-PLHCC, Human Metabolome Technologies, Yamagata, Japan) to remove macromolecules, and resuspended in 50 µL of ultra-pure water immediately before the metabolome analysis using CE-TOFMS. The detected peaks were annotated based on the m/z value and normalized migration time. The relative area value of each peak was calculated and used for the intergroup comparison.

Illumina adapters for metagenomics analysis

Forward Primer: TCGTCGGCAGCGTCAGATGTGTATAAGAGACAG

Reverse Primer: GTCTCGTGGGCTCGGAGATGTGTATAAGAGACAG

Illumina barcode sequences for metagenomics analysis

	Barcode	Sequences	Barcode	Sequences
CON_1	N701	TAAGGCGA	S517	GCGTAAGA
CON_2	N701	TAAGGCGA	S502	CTCTCTAT
CON_3	N701	TAAGGCGA	S503	TATCCTCT
CON_4	N701	TAAGGCGA	S504	AGAGTAGA
CON_5	N701	TAAGGCGA	S505	GTAAGGAG
CON_6	N701	TAAGGCGA	S506	ACTGCATA
CON_7	N701	TAAGGCGA	S507	AAGGAGTA
CON_8	N701	TAAGGCGA	S508	CTAAGCCT
DSS_1	N702	CGTACTAG	S517	GCGTAAGA
DSS_2	N702	CGTACTAG	S502	CTCTCTAT
DSS_3	N702	CGTACTAG	S503	TATCCTCT
DSS_4	N702	CGTACTAG	S504	AGAGTAGA
DSS_5	N702	CGTACTAG	S505	GTAAGGAG
DSS_6	N702	CGTACTAG	S506	ACTGCATA
DSS_7	N702	CGTACTAG	S507	AAGGAGTA

DSS_8	N702	CGTACTAG	S508	CTAAGCCT
D-ESM8_1	N703	AGGCAGAA	S517	GCGTAAGA
D-ESM8_2	N703	AGGCAGAA	S502	CTCTCTAT
D-ESM8_3	N703	AGGCAGAA	S503	TATCCTCT
D-ESM8_4	N703	AGGCAGAA	S504	AGAGTAGA
D-ESM8_5	N703	AGGCAGAA	S505	GTAAGGAG
D-ESM8_6	N703	AGGCAGAA	S506	ACTGCATA
D-ESM8_7	N703	AGGCAGAA	S507	AAGGAGTA
D-ESM8_8_	N703	AGGCAGAA	S508	CTAAGCCT

Sequencing outcome of metagenome analysis

After quality assessment in QIIME, a total of 629,440 quality-filtered sequences were obtained from the samples with a mean of 5473 ± 1030 (SD) sequences per sample, a maximum of 7197, and a minimum of 2860 reads.

Supplementary Table S1. Diet composition

% (w/w)	Control diet	8% ESM diet
Casein	20.0	16.3
β-corn starch	39.8	35.4
α-corn starch	13.2	13.2
Soybean oil	10.3	10.3
Sucrose	7.0	7.0
Cellulose	5.0	5.0
Vitamin mixture (AIN93G-MX)	3.5	3.5
Mineral mixture (AIN93G-MX)	1.0	1.0
L-cystine	0.30	0.30
ESM powder	0.0	8.0

Supplementary Table S2. Primer sequences

Human primers

Gene	Accession no	Primer sequence	Probe Set ID
IL1B	NM_000576	Forward GCCTGGACTTTCCTGTTGTC<br>Reverse AGAATGTGGGAGCGAATGAC	205067_at
CTGF	M92934	Forward TGCTCACTGACCTGCCTGTA<br>Reverse TTCACTTGCCACAAGCTGTC	209101_at
PDGFA	NM_002607	Forward TGTTTCTCCCTTACCCTGCTT<br>Reverse GGCACACCAACAACACAGAC	205463_s_at
EDN1	J05008	Forward CCGGCTAATGAAAGAGGTTG<br>Reverse TTGACAGGCAAAACAAAGCA	222802_at
GAPDH	AK026525	Forward CTCATGACCACAGTCCATGC<br>Reverse CTCATGACCACAGTCCATGC	217398_x_at

Mouse primers

Gene	Accession no	Primer sequence	Probe Set ID
Actβ	NM_007393	Forward GACGGCCAGGTCATCACTAT<br>Reverse CTTCTGCATCCTGTCAGCAA	M12481_3_at
Cav1	NM_007616	Forward CTCTGCCCTTGGGGATATTT<br>Reverse GCCATGCCAGTGTCTGTTTT	1449145_a_at
Ccl6	NM_009139	Forward CAAGTAAGAACCTGCACACCA<br>Reverse TTGGGGTAAACCAGGGCTAT	1417266_at
Ccl9	NM_011338	Forward GCCCTATTTTGCCCCTTTAG<br>Reverse TCCCACAGACCACTCTCACA	1417936_at
Ccl11	NM_011330	Forward CCAAGGACTTGGCTTCATGT<br>Reverse CTCGTCCCATTGTGTTCCTC	1417789_at
Cd14	NM_009841	Forward GTCAGGAACTCTGGCTTTGC<br>Reverse ATCAGGGGTCAAGTTTGCTG	1417268_at
Cfh	NM_009888	Forward AGATTCACCGCCATTTCGTA<br>Reverse TGCTTTTGCATTTTGGCAAT	1423153_x_at
Col18a1	NM_001109991	Forward CAGGCTCCTGGAACAGAAAG<br>Reverse GATTGTATCCCAGCCAGAGC	1418237_s_at
Ctgf	NM_010217	Forward CAAACAAATGCTGTGCAGGT<br>Reverse AGCAAGCACTTCCTGGTAGG	1416953_at
Cxcl1	NM_008176	Forward GCTGGGATTCACCTCAAGAA<br>Reverse AGGTGCCATCAGAGCAGTCT	1441855_x_at
Cxcl9	NM_008599	Forward ACGGAGATCAAACCTGCCTA<br>Reverse GATTCAGGGTGCTTGTTGGT	1418652_at

Cxcl13	NM_018866	Forward CGGTATTCTGGAAGCCCATT<br>Reverse GCTTGGGGAGTTGAAGACAG	1417851_at
Edn1	NM_010104	Forward CATTCCAAGAAAGGCTGAGG<br>Reverse GCCTGAGTCAGACACGAACA	1451924_a_at
Fn1	NM_010233	Forward TGACTGTAGTGTGCCCCAAG<br>Reverse GCACTGACTGCTCTTCCAGA	1437218_at
Fpr1	NM_013521	Forward TCCATTGTTGCCATTTGCTA<br>Reverse GCTGTTGAAGAAAGCCAAGG	1450808_at
Hspb1	NM_013560	Forward GAAGAAAGGCAGGACGAACA<br>Reverse CGAAAGTAACCGGAATGGTG	1425964_x_at
Id4	NM_031166	Forward GTTCACGAGCATTCACCGTA<br>Reverse GGTTGGATTCACGATTGCTC	1423259_at
Igfbp3	NM_008343	Forward GCCTGGTAAGAGCATGGAGA<br>Reverse TCCAGACACAGGCTCCTTTC	1423062_at
Il1β	NM_008361	Forward TGAAGGAGCTCCCTTGTCAT<br>Reverse GGTAAGTGGTTGCCCATCAG	1449399_a_at
Il17a	NM_010552	Forward CCTAAGAAACCCCCACGTTT<br>Reverse TGAATCCACATTCCTTGCTG	1421672_at
Il6	NM_031168	Forward GCCAGAGTCCTTCAGAGAGATACA<br>Reverse CTTGGTCCTTAGCCACTCCTTC	1450297_at
Klf9	NM_010638	Forward TCGCAGGTGTTCAAAAAGAA<br>Reverse AGCTTAAAGCCAGGGTGTCA	1428288_at
Lbp	NM_008489	Forward GTGAACCTGTTCCAGGCATT<br>Reverse GGACATTGGCACCCAAGTAT	1448550_at
Lcn2	NM_008491	Forward CACGGACTACAACCAGTTCG<br>Reverse TCCTTGAGGCCCAGAGACT	1427747_a_at
Mmp7	NM_010810	Forward CCCGGTACTGTGATGTACCC<br>Reverse TTCTGAATGCCTGCAATGTC	1449478_at
Mmp9	NM_013599	Forward GCTCTCTACTGGGCGTTAGG<br>Reverse AGGAGTCTGGGGTCTGGTTT	1416298_at
Nos2	NM_010927	Forward ATTGCTCCCTTCCGAAGTTT<br>Reverse CACTCTCTTGCGGACCATCT	1420393_at
Nrp2	NM_001077403	Forward CAGCGACACCCATAGATTCC<br>Reverse TCACCACTCCTGCATTTTCA	1447343_at
Palld	NM_001081390	Forward CAGGTGTTCCTGTGCAGTGT<br>Reverse CAAGCCCAGCAAATTAGGAA	1433768_at
Pdgfd	NM_027924	Forward TGGATCATCATGAGCGATGT<br>Reverse CCATGGCACTAACAAAGCAA	1426319_at
Pik3ca	NM_008839	Forward GTTGTGGTGATGATGGTGGA<br>Reverse GATTCTTGGAGCCTGGAGTG	1440054_at

Reg3β	NM_011036	Forward GGCTTCATTCTTGTCCTCCA<br>Reverse AGATGGGTTCCTCTCCCAGT	1416297_s_at
Reg3γ	NM_011260	Forward GCCCTCAGGACATCTTGTGT<br>Reverse CACATCAGCATTGCTCCACT	1448872_at
Rplp1	NM_018853	Forward ATCTACTCCGCCCTCATCCT<br>Reverse CAGATGAGGCTCCCAATGTT	1416277_a_at
S100a8	NM_013650	Forward GGAAATCACCATGCCCTCTA<br>Reverse ATCACCATCGCAAGGAACTC	1419394_s_at
S100a9	NM_009114	Forward CAGCATAACCACCATCATCG<br>Reverse GTCCTGGTTTGTGTCCAGGT	1448756_at
Tgfb2	NM_009367	Forward CCTTTTCTGCGTCAGTGTGA<br>Reverse GCTTTTGAACGGCAAAGAGA	1450923_at
Tgfb3	NM_009368	Forward GCACTGCCTGGAATTAAGGA<br>Reverse AAGAAGGAAGGCAGGAGGAG	1417455_at
Timp2	NM_011594	Forward CCAGGTCTCTCTGGCACTGT<br>Reverse TAGGCCGGCTACACAGTCTT	1454677_at
Timp3	NM_011595	Forward GCCATTCTCAATCCCTGGTA<br>Reverse TGGCCAGTACTTCTCACCAA	1449335_at
Tlr4	NM_021297	Forward ATGGAAAAGCCTCGAATCCT<br>Reverse CTCTCGGTCCATAGCAGAGC	1418162_at
Tpm1	NM_001164248	Forward GATGCTGACCGGAAGTATGA<br>Reverse CCTGAGCCTCCAGTGACTTC	1423049_a_at
Tpm2	NM_009416	Forward TGGCAAAGTTGGAGAAAACC<br>Reverse CCCGAGGAGGCAACTATGTA	1425028_a_at

Bacterial primers

Target Organism	Primer sequence
Total bacteria	Forward ACTCCTACGGGAGGCAGCAG<br>Reverse GTATTACCGCGGCTGCTGGCA
E. coli	Forward CATGCCGCGTGTATGAAGAA<br>Reverse CGGGTAACGTCAATGAGCAAA
SFB	Forward AGGAGGAGTCTGCGGCACATTAGC<br>Reverse TCCCCACTGCTGCCTCCCGTAG

Supplementary Table S3

Food (g/day) and water (ml/day) intake	CON	DSS	D-ESM8
Food (before DSS)	3.7±0.1	3.8±0.2	3.5±0.1
Food (after DSS)	3.9±0.2	3.5±0.3	3.3±0.1
Water (before DSS)	3.0±0.1	2.9±0.2	2.9±0.1
Water (after DSS)	2.9±0.1	2.7±0.1	2.7±0.1
**Tissue weight (g)**	CON	DSS	D-ESM8
Liver	0.99±0.02 a	1.07±0.04 ab	1.09±0.02 b
Spleen	0.07±0.00 a	0.10±0.01 b	0.10±0.01 b
Mesenteric fat	0.19±0.02 a	0.06±0.01 b	0.07±0.01 b
**Hepatic lipids**	CON	DSS	D-ESM8
TG (mg/g liver)	10.6±0.6 a	7.6±0.3 b	8.3±0.7 b
TC (mg/g liver)	4.0±0.1	4.4±0.1	3.8±0.2
HDL cholesterol (mg/g liver)	3.0±0.1 a	3.4±0.1 b	3.0±0.1 a
NEFA (μEq/g liver)	10.1±0.4	10.1±0.3	10.0±0.2

Supplementary Table S4. Number of differentially expressed genes in colon

	**DSS vs. CON**	**D-ESM8 vs. DSS**
Increase	591	327
Decrease	615	285
Total	1206	612

Functional category (differentially expressed genes in colon - DSS vs. CON)

**Term**	***p*-value**
Granulocyte adhesion and diapedesis	5.32E-07
Dendritic cell maturation	1.09E-06
Hepatic fibrosis / hepatic stellate cell activation	3.61E-06
Inhibition of matrix metalloproteases	5.02E-06
Leukocyte extravasation signaling	8.99E-06
Agranulocyte adhesion and diapedesis	1.47E-05
Complement system	4.55E-05
Antigen presentation pathway	6.34E-05
Crosstalk between dendric cells and natural killer cells	6.52E-05
Atherosclerosis signaling	1.68E-04

Functional category (differentially expressed genes in colon – D-ESM8 vs. DSS)

**Term**	***p*-value**
Inhibition of matrix metalloproteases	2.97E-04
RhoA signaling	3.05E-04
Agranulocyte adhesion and diapedesis	3.62E-04
Cellular effects of sildenafil (Viagra)	5.06E-04
Granulocyte adhesion and diapedesis	8.27E-04
Regulation of actin-based motility by Rho	8.63E-04
Nicotine degradation II	2.43E-03
Neuroprotective role of THOP1 in alzheimer's diseases	2.63E-03
Estrogen biosynthesis	3.50E-03
Cdc42 signaling	3.75E-03

Supplementary Table S5. Number of differentially expressed genes in liver

	**DSS vs. CON**	**D-ESM8 vs. DSS**
Increase	1037	380
Decrease	596	565
Total	1633	945

Functional category (differentially expressed genes in liver - DSS vs. CON)

**Term**	***p*-value**
Acute phase response signaling	8.44E-06
Altered T cell and B cell signaling in rheumatoid arthritis	1.23E-05
TREM1 signaling	2.44E-05
Estrogen-mediated S-phase entry	3.56E-05
NF-κB signaling	3.66E-05
Communication between innate and adaptive immune cells	4.82E-05
Dendritic cell maturation	1.23E-04
Role of macrophages, fibroblasts and endothelial cells in rheumatoid arthritis	1.28E-04
IL-10 signaling	1.37E-04
IL-6 signaling	1.82E-04

Functional category (differentially expressed genes in liver – D-ESM8 vs. DSS)

**Term**	***p*-value**
Acute phase signaling	2.43E-03
Role of JAK1, JAK2 and TYK2 in interferon signaling	2.54E-03
Oncostatin M signaling	4.09E-03
Phenylethylamine degradation I	6.52E-03
LPS-stimulated MAPK signaling	6.71E-03
DNA methylation and transcriptonal repression signaling	7.25E-03
Toll-like receptor signaling	7.87E-03
Interferon signaling	9.33E-03
IL-6 signaling	1.24E-02
Polyamine regulation in colon cancer	1.32E-02

Supplementary Table S6. Number of differentially expressed proteins in liver

	**DSS vs. CON**	**D-ESM8 vs. DSS**
Increase	71	130
Decrease	117	51
Total	188	181

Functional category (differentially expressed proteins in liver - DSS vs. CON)

**Term**	***p*-value**
Superpathway of citrulline metabolism	3.05E-05
Superpathway of methionine degradation	4.13E-05
Urea cycle	4.9E-05
Aspartate degradation II	4.9E-05
Acute phase response signaling	5.58E-05
Valine degradation I	8.94E-05
Gluconeogenesis I	8.94E-05
Glycine Betaine degradation	2.82E-04
Phenylalanine degradation I (Aerobic)	5.54E-04
Glutamate degradation II	5.54E-04

Functional category (differentially expressed proteins in liver – D-ESM8 vs. DSS)

**Term**	***p*-value**
Arginine biosynthesis IV	1.34E-07
Urea cycle	3.99E-07
Phenylalanine degradation IV (Mammalian, via side chain)	4.06E-07
Gluconeogenesis I	2.56E-07
Glutaryl-CoA degradation	8.34E-06
Mitochondrial dysfunction	1.05E-05
Fatty acid β-oxidation I	2.2E-05
Superpathway of citrulline metabolism	2.45E-05
Aspartate degradation II	4.15E-05
Glycolysis I	5.66E-05

Supplementary Table S7. Concentration of metabolites in plasma

**Metabolite（μM）**	**CON**	**DSS**	**D-ESM8**
Citrate	1.4±0.0 $\times10^2$ a	1.2±0.0 $\times10^2$ b	1.6±0.1 $\times10^2$ c
Cis-Aconitate	7.2±0.2 a	6.0±0.4 b	8.3±0.4 a
Isocitrate	7.3±0.3 a	6.1±0.2b	8.4±0.3 a
2-Oxoglutarate	51.1±4.5 a	36.4±5.1 b	57.5±4.4 a
Succinate	30.5±1.7 a	21.2±1.6 b	34.7±2.3 a
Fumarate	13±1.1 ab	10.2±2.3 a	17.2±1.6 b
Malate	80.9±6.0 a	60.7±8.9 b	96.7±8.7 a
3PG	3.2±0.4a	1.8±0.3b	2.6±0.4ab
Hydroxyproline	31.2±2.1 a	19.3±1.5 b	38.0±3.6 a
5-Hydroxylysine	7.9±0.7 $\times10^{-1}$ a	6.3±0.4 $\times10^{-1}$ b	13±1 $\times10^{-1}$ c
Trp	71.9±4.9 a	45.3±6.3 b	71.7±5.6 a
Gly	2.9±0.1 $\times10^2$ a	2.7±0.2 $\times10^2$ a	3.6±0.3 $\times10^2$ b
Cystine	8.4±0.8a	8.5±1.1a	11.1±0.3b
beta-Ala	3.5±0.2 a	2.6±0.2 b	3.2±0.3 ab
Urea	8.9±0.4 $\times10^3$ a	7.2±0.6 $\times10^3$ b	9.5±0.7 $\times10^3$ a

Supplementary Table S8. Concentration of metabolites in liver

**Metabolite（nmol/g）**	**CON**	**DSS**	**D-ESM8**
Citrate	1.3±0.1 $\times 10^2$	5.7±2.5 $\times 10^2$	4.1±1.9 $\times 10^2$
Cis-Aconitate	7.3±0.8	33.9±16.8	24.1±12.9
Isocitrate	15.1±0.9	44.8±19.1	36.1±13.9
Succinate	2.7±0.3 $\times 10^2$ a	2.1±0.3 $\times 10^2$ ab	1.5±0.1 $\times 10^2$ b
Fumarate	1.9±0.1 $\times 10^2$ a	4.7±0.4 $\times 10^2$ b	4.4±0.3 $\times 10^2$ b
G1P	1.1±0.1 $\times 10^2$ ab	1.5±0.2 $\times 10^2$ a	1.0±0.1 $\times 10^2$ b
F1,6P	2.6±0.3 $\times 10^2$ a	4.4±0.3 $\times 10^2$ b	3.6±0.3 $\times 10^2$ c
NAD^+	39.9±1.3 a	29.8±1.4 b	30.7±0.7 b
FAD	61.2±2.1 a	53.9±1.4 b	60.3±1.7 a

Supplementary Table S9. Variations in the cecal microbiota profiles

Phylum	Class	Order	Family	Genus	Species	CON		DSS		D-ESM8	
						Mean	SE	Mean	SE	Mean	SE
Actinobacteria	Actinobacteria	Actinomycetales	Corynebacteriaceae	Corynebacterium		0.0000	0.0000	0.0064	0.0063	0.0009	0.0014
			Microbacteriaceae	Microbacterium		0.0000	0.0000	0.0028	0.0048	0.0000	0.0000
			Micrococcaceae	Micrococcus	Other	0.0000	0.0000	0.0000	0.0000	0.0034	0.0050
				Rothia	dentocariosa	0.0000	0.0000	0.0000	0.0000	0.0027	0.0031
			Propionibacteriaceae	Propionibacterium	acnes	0.0021	0.0026	0.0166	0.0141	0.0046	0.0032
		Bifidobacteriales	Bifidobacteriaceae	Bifidobacterium	Other	0.0024	0.0022	0.0025	0.0025	0.0000	0.0000
					pseudolongum	0.1859	0.0334	0.2164	0.1382	0.0247	0.0142
	Coriobacteriia	Coriobacteriales	Coriobacteriaceae			0.0000	0.0000	0.0000	0.0000	0.0041	0.0035
				Adlercreutzia		0.0039	0.0030	0.0555	0.0358	0.0262	0.0080
Bacteroidetes	Bacteroidia	Bacteroidales	Bacteroidaceae	Bacteroides	Other	0.0180	0.0111	0.0349	0.0204	0.0574	0.0158
						22.5359	1.4623	21.4131	5.9791	19.3097	4.3965
					caccae	0.3717	0.0356	0.3172	0.0996	0.3117	0.0566
			Porphyromonadaceae	Parabacteroides		22.5411	1.1132	3.1693	1.6198	9.0597	2.5057
			Prevotellaceae	Prevotella	melaninogenica	0.0000	0.0000	0.0000	0.0000	0.0007	0.0016
			Rikenellaceae			4.1915	0.4307	8.9805	2.4588	24.6949	3.1697
			S24-7			17.3383	1.7678	0.5994	0.2788	1.7148	0.6593
	Cytophagia	Cytophagales	Cytophagaceae	Hymenobacter		0.0000	0.0000	0.0024	0.0037	0.0015	0.0023
	Flavobacteriia	Flavobacteriales	Flavobacteriaceae			0.0000	0.0000	0.0018	0.0022	0.0007	0.0017
			[Weeksellaceae]	Chryseobacterium		0.0000	0.0000	0.0027	0.0033	0.0005	0.0011
				Cloacibacterium		0.0000	0.0000	0.0015	0.0031	0.0000	0.0000
	Sphingobacteriia	Sphingobacteriales				0.0000	0.0000	0.0086	0.0080	0.0000	0.0000
			Sphingobacteriaceae	Sphingobacterium	multivorum	0.0004	0.0008	0.0228	0.0170	0.0012	0.0015
Chlamydiae	Chlamydiia	Chlamydiales	Parachlamydiaceae			0.0000	0.0000	0.0016	0.0019	0.0000	0.0000
Cyanobacteria	ML635J-21					0.0000	0.0000	0.0005	0.0010	0.0008	0.0018

Deferribacteres	Deferribacteres	Deferribacterales	Deferribacteraceae	Mucispirillum	schaedleri	2.2182	0.6877	7.6689	2.7778	14.0131	1.9433
		Bacillales	Staphylococcaceae	Staphylococcus	Other	0.0000	0.0000	0.0035	0.0044	0.0000	0.0000
			Other	Other	Other	0.0009	0.0014	0.1427	0.0416	0.0496	0.0187
			Carnobacteriaceae	Granulicatella		0.0000	0.0000	0.0000	0.0000	0.0033	0.0047
				Other	Other	0.0000	0.0000	0.0023	0.0037	0.0000	0.0000
			Enterococcaceae	Enterococcus	Other	0.0000	0.0000	0.0287	0.0228	0.0000	0.0000
	Bacilli	Lactobacillales				0.0000	0.0000	0.0024	0.0024	0.0011	0.0019
			Lactobacillaceae	Lactobacillus		0.0617	0.0214	0.1918	0.0658	0.0484	0.0269
				Lactococcus		0.0004	0.0008	0.0251	0.0192	0.0019	0.0024
			Streptococcaceae	Streptococcus		0.0000	0.0000	0.0033	0.0031	0.0036	0.0053
					anginosus	0.0000	0.0000	0.0000	0.0000	0.0009	0.0013
		Turicibacterales	Turicibacteraceae	Turicibacter		0.1573	0.0564	0.2044	0.0772	0.3494	0.1060
			Other	Other	Other	2.5944	0.5022	0.1294	0.0597	0.0240	0.0153
						9.2744	1.1197	2.1820	0.4611	5.5986	1.1155
Firmicutes			Christensenellaceae			0.0679	0.0178	0.0567	0.0226	0.1310	0.0333
				Other	Other	0.0020	0.0022	0.0030	0.0026	0.0017	0.0019
			Clostridiaceae			0.3232	0.1378	1.7541	0.1818	0.6341	0.2727
				Clostridium		0.0000	0.0000	0.0026	0.0043	0.0000	0.0000
				SMB53		0.0000	0.0000	0.0068	0.0048	0.0008	0.0012
	Clostridia	Clostridiales	Dehalobacteriaceae	Dehalobacterium		0.1479	0.0253	0.0707	0.0357	0.0890	0.0198
				Other	Other	0.0078	0.0062	0.0039	0.0031	0.0403	0.0320
						3.1216	0.5261	0.4141	0.1358	0.5075	0.1677
				Blautia		0.0000	0.0000	0.0000	0.0000	0.0007	0.0016
			Lachnospiraceae	Coprococcus		0.2424	0.0595	0.0698	0.0300	0.2878	0.1099
				Dorea		0.1207	0.0348	0.0953	0.0645	0.4294	0.1043
				Epulopiscium		0.0000	0.0000	0.0010	0.0015	0.0000	0.0000
				[Ruminococcus]	gnavus	0.3653	0.0943	1.4203	0.4865	1.1391	0.2634

			Peptococcaceae			0.0012	0.0019	0.0006	0.0012	0.0050	0.0051
				rc4-4		0.3639	0.0857	0.1098	0.0614	0.0763	0.0372
			Peptostreptococcaceae			0.0000	0.0000	0.0376	0.0091	0.0103	0.0103
				Other	Other	0.0009	0.0014	0.0068	0.0059	0.0027	0.0027
						4.5710	0.4871	1.6391	0.5915	3.3619	0.4399
			Ruminococcaceae	Anaerotruncus		0.0000	0.0000	0.0412	0.0290	0.0207	0.0087
				Oscillospira		4.3067	0.3838	3.7424	0.7753	4.7732	0.9402
				Ruminococcus		0.6801	0.0971	0.1714	0.0607	0.4649	0.0781
			Veillonellaceae	Veillonella	dispar	0.0000	0.0000	0.0000	0.0000	0.0012	0.0020
			[Mogibacteriaceae]			0.0148	0.0100	0.0052	0.0050	0.0644	0.0233
		SHA-98				0.0010	0.0023	0.0000	0.0000	0.0000	0.0000
				Other	Other	0.0004	0.0009	0.0050	0.0037	0.0007	0.0012
						0.0550	0.0166	1.0636	0.7394	0.3574	0.1382
	Erysipelotrichi	Erysipelotrichales	Erysipelotrichaceae	Allobaculum		0.0163	0.0080	0.0246	0.0158	0.1169	0.1102
				Coprobacillus		0.0000	0.0000	0.0140	0.0078	0.0019	0.0018
				[Eubacterium]	dolichum	0.0011	0.0017	0.0562	0.0330	0.0203	0.0087
OD1	ZB2					0.0010	0.0023	0.0428	0.0337	0.0027	0.0028
						0.0000	0.0000	0.0012	0.0024	0.0000	0.0000
		Caulobacterales	Caulobacteraceae			0.0000	0.0000	0.0099	0.0085	0.0000	0.0000
		RF32				0.0000	0.0000	0.0000	0.0000	0.0011	0.0017
			Brucellaceae	Ochrobactrum		0.0000	0.0000	0.0018	0.0022	0.0000	0.0000
			Hyphomicrobiaceae	Pedomicrobium		0.0000	0.0000	0.0000	0.0000	0.0007	0.0012
Proteobacteria	Alphaproteobacteria		Methylobacteriaceae	Methylobacterium		0.0000	0.0000	0.0027	0.0044	0.0000	0.0000
		Rhizobiales				0.0000	0.0000	0.0012	0.0025	0.0000	0.0000
			Phyllobacteriaceae	Phyllobacterium		0.0000	0.0000	0.0075	0.0070	0.0000	0.0000
			Rhizobiaceae	Agrobacterium		0.0000	0.0000	0.0027	0.0029	0.0000	0.0000
		Sphingomonadales	Erythrobacteraceae			0.0000	0.0000	0.0000	0.0000	0.0008	0.0017

					0.0000	0.0000	0.0040	0.0061	0.0000	0.0000
		Sphingomonadaceae	Novosphingobium		0.0000	0.0000	0.0000	0.0000	0.0009	0.0019
			Sphingomonas		0.0000	0.0000	0.0265	0.0193	0.0068	0.0071
				yabuuchiae	0.0000	0.0000	0.0137	0.0117	0.0000	0.0000
		Alcaligenaceae	Sutterella		0.8019	0.1722	0.6554	0.2747	1.9766	0.4030
			Other	Other	0.0000	0.0000	0.0123	0.0080	0.0000	0.0000
		Comamonadaceae			0.0018	0.0024	0.0233	0.0175	0.0144	0.0114
			Roseateles	depolymerans	0.0000	0.0000	0.0149	0.0142	0.0000	0.0000
Betaproteobacteria	Burkholderiales		Variovorax		0.0000	0.0000	0.0016	0.0019	0.0000	0.0000
					0.0000	0.0000	0.0037	0.0046	0.0000	0.0000
		Oxalobacteraceae	Janthinobacterium	Other	0.0000	0.0000	0.0011	0.0016	0.0000	0.0000
			Ralstonia		0.0000	0.0000	0.0011	0.0016	0.0000	0.0000
	Neisseriales	Neisseriaceae			0.0000	0.0000	0.0101	0.0089	0.0000	0.0000
	SBla14				0.0000	0.0000	0.0014	0.0020	0.0000	0.0000
Deltaproteobacteria	Myxococcales				0.0000	0.0000	0.0000	0.0000	0.0009	0.0021
	Alteromonadales	Shewanellaceae	Shewanella		0.0000	0.0000	0.0068	0.0059	0.0000	0.0000
			Other	Other	0.0000	0.0000	0.0040	0.0041	0.0008	0.0013
	Enterobacteriales	Enterobacteriaceae			0.0559	0.0337	39.0917	11.4644	6.0227	2.1925
			Escherichia	coli	0.0000	0.0000	0.0684	0.0256	0.0047	0.0035
					0.0004	0.0008	0.0231	0.0144	0.0025	0.0029
			Acinetobacter	johnsonii	0.0000	0.0000	0.0158	0.0137	0.0033	0.0042
Gammaproteobacteria		Moraxellaceae		rhizosphaerae	0.0005	0.0011	0.1583	0.1400	0.0004	0.0008
	Pseudomonadales		Enhydrobacter		0.0000	0.0000	0.0000	0.0000	0.0014	0.0018
		Pseudomonadaceae	Pseudomonas		0.0005	0.0011	0.0212	0.0153	0.0018	0.0020
				viridiflava	0.0000	0.0000	0.0000	0.0000	0.0007	0.0016
	Xanthomonadales	Xanthomonadaceae			0.0000	0.0000	0.0161	0.0140	0.0023	0.0032
			Stenotrophomonas		0.0000	0.0000	0.0006	0.0012	0.0040	0.0032

TM6	SJA-4					0.0000	0.0000	0.0011	0.0024	0.0000	0.0000
TM7	TM7-3	EW055				0.0000	0.0000	0.0053	0.0066	0.0008	0.0017
		Anaeroplasmatales	Anaeroplasmataceae	Anaeroplasma		0.0155	0.0123	0.0000	0.0000	0.0664	0.0340
Tenericutes	Mollicutes	Mycoplasmatales	Mycoplasmataceae	Mycoplasma		0.0000	0.0000	0.0000	0.0000	0.0008	0.0013
		RF39				0.0119	0.0093	0.0000	0.0000	0.0271	0.0172
Verrucomicrobia	Verrucomicrobiae	Verrucomicrobiales	Verrucomicrobiaceae	Akkermansia	muciniphila	3.1633	1.3024	3.4116	1.9935	3.8547	1.4871
WPS-2						0.0000	0.0000	0.0000	0.0000	0.0009	0.0014
Unassigned						0.0379	0.0111	0.0805	0.0207	0.1463	0.0344

The blank is the unassigned taxa currently.

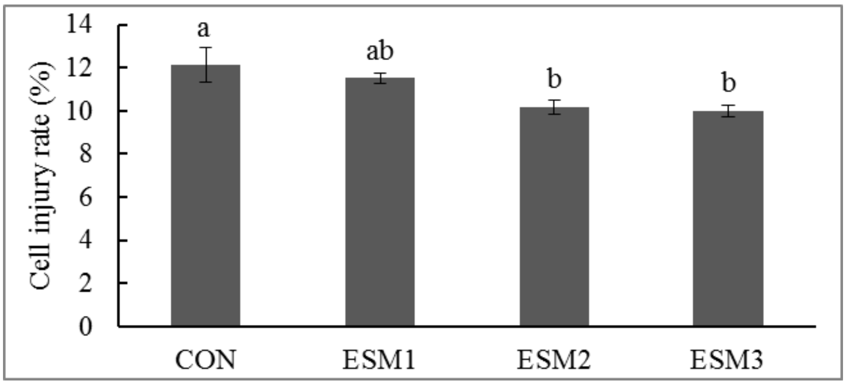

Suppl. Fig. S1 Caco-2 cell injury rate under LPS stimulation

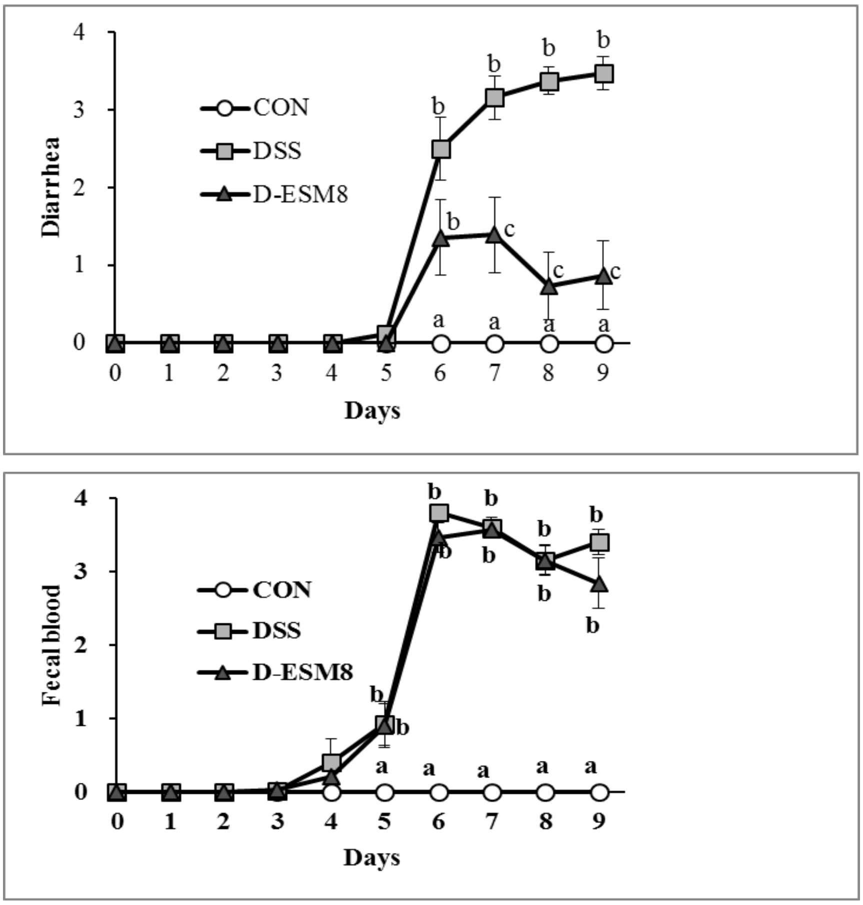

Suppl. Fig. S2 Changes in diarrhea and fecal blood of the mice.

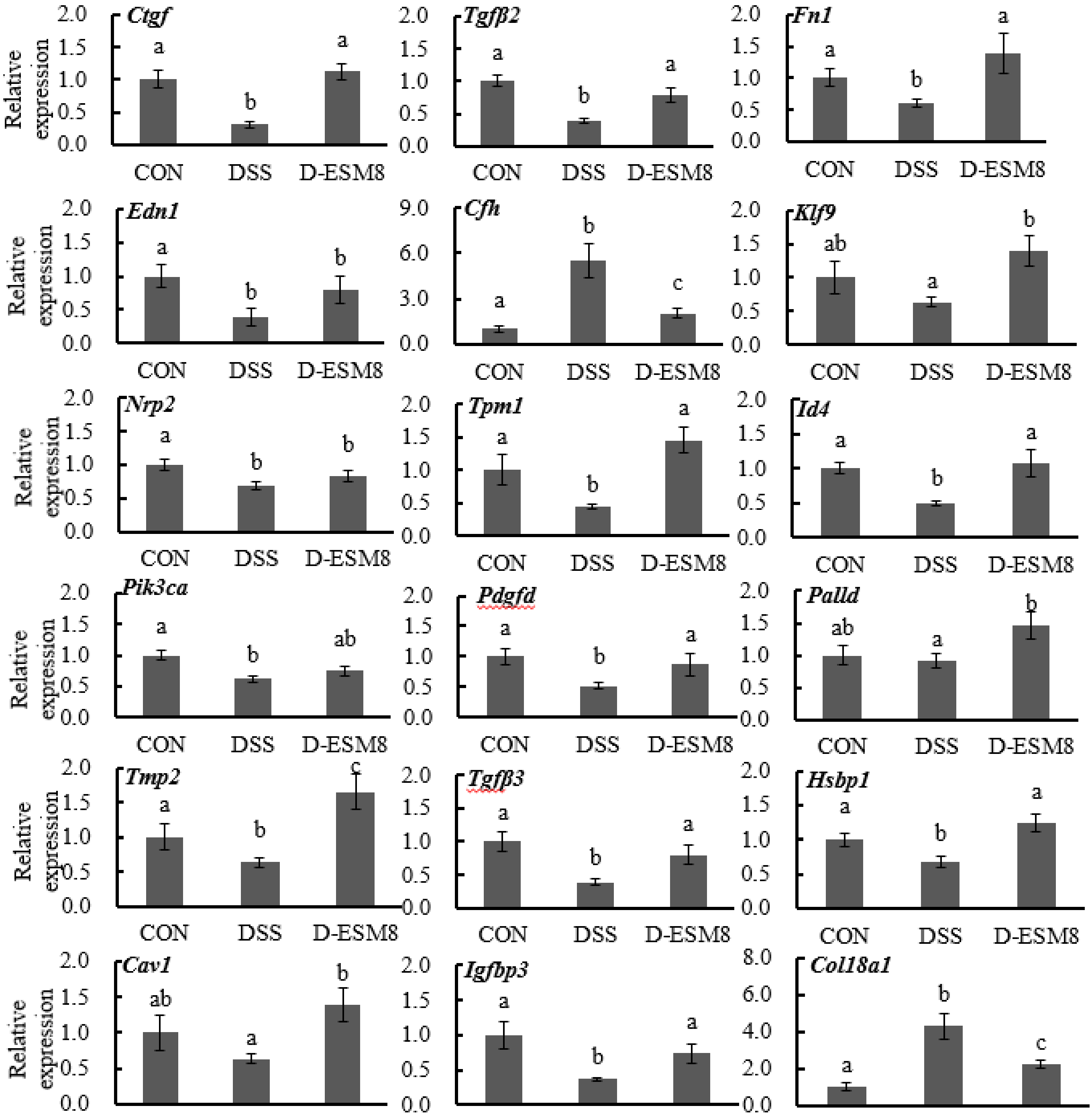

Suppl. Fig. S3 Expression of genes related to intestinal epithelial proliferation and restoration

DSS vs. CON   D-ESM8 vs. DSS

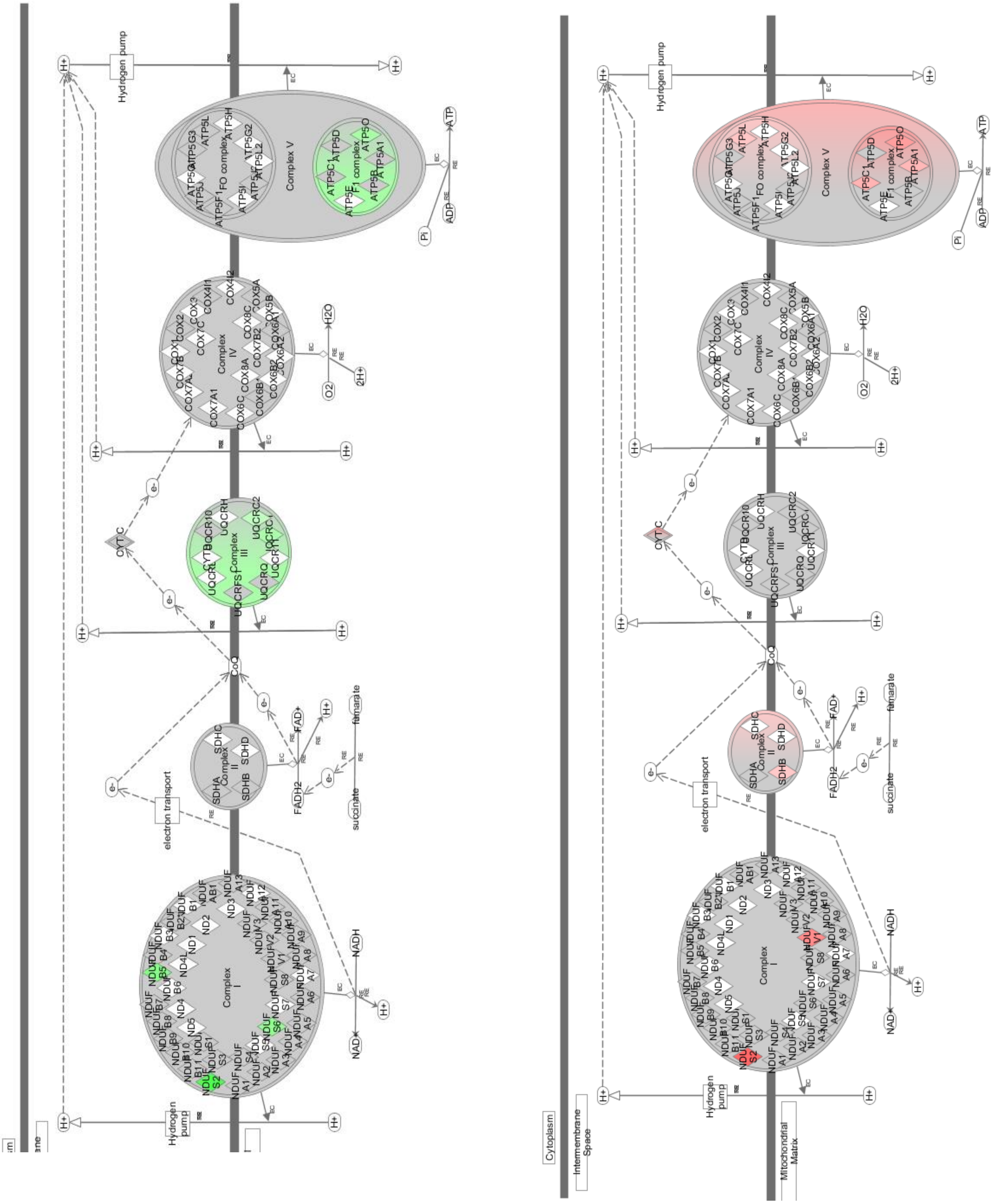

Suppl. Fig. S4 Expression of proteins related to electron transport chain and oxidative phosphorylation in liver according to IPA. Red and green indicate up- and down-regulation, with the more intense color indicating a greater change.
